# A Cis‐Regulatory Duplication in a Hox Hotspot Implicated in Mimetic Convergence in the Bumble Bee *Bombus flavifrons*

**DOI:** 10.1111/mec.70539

**Published:** 2026-09-07

**Authors:** Tunç Dabak, Jeffrey D. Lozier, Cecil Smith, Brandon Meadows, Jonathan Berenguer Uhuad Koch, Heather M. Hines

**Affiliations:** ^1^ Department of Biology Pennsylvania State University University Park Pennsylvania USA; ^2^ Department of Biological Sciences The University of Alabama Tuscaloosa Alabama USA; ^3^ Pacific Cooperative Studies Unit The University of Hawai‘i, Mānoa Honolulu Hawaii USA; ^4^ Department of Entomology Pennsylvania State University University Park Pennsylvania USA

**Keywords:** bumble bee, evolutionary genetics, Hox, introgression, mimicry

## Abstract

Several species of North American bumble bees spanning the Pacific Coastal and Rocky Mountain regions converge onto distinct mimetic abdominal colour forms for each region by switching abdominal coloration from black to red. Previous genome‐wide association studies (GWAS) of red and black transitions in two mimics ( *Bombus melanopygus* and *Bombus vancouverensis* ) revealed that black forms were generated by independently deleting a portion of the same cis‐regulatory region near the Hox gene *Abdominal‐B* (*Abd‐B*). Here, we test the genetic basis of these mimetic colour forms in a third co‐mimic, *Bombus flavifrons* , that has continuous variation in red and black that is shifted posteriorly one segment compared to its co‐mimics. Using genome‐wide association of red and black forms, we identified a structural variant < 50 bp away from the deletions in *B. melanopygus* and *B. vancouverensis* that was strongly associated with the colour phenotype. Sequencing across mimicry zones and closely related taxa revealed that all red forms of *B. flavifrons* and monomorphic red close relative *Bombus centralis* have a 319 bp tandem duplication at this locus that has extensive modification to the duplicated copy. Black forms of *B. flavifrons* from the Cascades also have this duplication but without the modifications, while black forms in the western Rockies mostly lack this duplication, similar to ancestral black forms. This suggests independent mechanisms may regulate the black phenotypes in different populations and that ancestral sorting of variation and/or adaptive introgression generated these phenotypes. This study strengthens support for this *Abd‐B* cis‐regulatory region being a hotspot for regulating abdominal coloration in bumble bees, and features the role of regulatory region duplication in creating novel phenotypes.

## 1 Introduction

Systems in which similar traits evolve independently in multiple lineages are particularly informative for understanding evolutionary genetics as they provide natural replicates to make predictions on which genes, parts of genes, and components of developmental pathways are most likely to be targeted in evolution. Colour polymorphisms, in particular, are powerful tools for addressing these questions because they are readily visible phenotypes under strong selection and thus are both diverse and highly convergent (Hoekstra et al. 2006; Martin and Orgogozo 2013).

Among examples of evolutionary genetics insights involving colour convergence, research on the repeated evolution of melanic coloration in mice has revealed the melanocortin‐1 receptor (Mc‐1r) protein is frequently modified due to its low pleiotropy, but colour can be regulated by more highly pleiotropic genes which are instead targeted in cis‐ (Nachman et al. 2003; Kingsley et al. 2009; Linnen et al. 2009). In convergent white lizards, different parts of the same Mc‐1r protein are targeted, showing there are many ways within proteins to shift function (Rosenblum et al. 2010). In *Drosophila*, similar melanic colour patterns typically evolve by modifying melanin pathway genes in cis‐ that alters their binding to upstream body region genes, but different species and populations may target different cis‐regulatory regions or genes in the same pathway to the same effect (Wittkopp, Carroll, et al. 2003; Wittkopp, Williams, et al. 2003; Kronforst et al. 2012).

Müllerian mimicry, whereby unrelated defended species repeatedly converge on similar warning colour patterns in response to predator‐mediated selection, provides some of the most striking examples of colour‐based convergence. Comimetic *Heliconius* butterfly species have converged onto multiple regional mimicry complexes across the Neotropics, which has led to divergence within species as populations cross different mimicry zones. *Heliconius* wing pattern diversity and convergence was found to be generated by modification of the same three major developmental loci, revealing these to be evolutionary hotspots for generating variation (Kronforst and Papa 2015; McMillan et al. 2020). The repeated phenotypes arise through different regulatory mutations in the same genes in distantly related species (Kronforst and Papa 2015; Supple et al. 2013), whereas closely related taxa often share alleles through adaptive introgression or sorting of ancestral polymorphisms (Pardo‐Diaz et al. 2012; Kronforst 2012). This system thus informs how alleles both arise and sort across populations and species.

Bumble bees (*Bombus* Latrielle) represent an analogous system for investigating these questions due to their exceptional colour diversity that is largely a result of mimicry. The ~270 bumble bee species display over 400 different dorsal segmental body patterns involving combinations of black, red, yellow and white setae (Cameron et al. 2007; Williams 2007; Rapti et al. 2014). Although factors such as thermoregulatory adaptation may play a role (Stiles 1979; Pekkarinen and Teräs 1993; Williams 2007), Müllerian mimicry has been proposed as the principal driver of colour pattern diversification in bumble bees. Aposematic colour patterns function as a shared warning signal advertising a venomous sting, which leads predators to avoid these patterns after initial exposure to bumble bees (Morgan 1896; Brower and Westcott 1960; Evans and Waldbauer 1982). This process has resulted in the evolution of at least 24 distinct geographic colour pattern complexes worldwide (Williams 2007). Like *Heliconius*, these mimicry regions contain multiple species from different evolutionary lineages, thus involve repeated instances of convergence, but this also generates considerable intraspecific diversity as species cross these zones. When several species span neighbouring mimicry zones, they each undergo similar colour shifts to conform to local complexes, providing replication ideal for studying the genetic mechanisms that produce adaptive coloration and for understanding the processes promoting phenotypic diversification and evolution (Hines and Rahman 2019).

One replicated set of colour transitions in bumble bees occurs in species spanning the Pacific Coastal and Rocky Mountain mimicry complexes of western North America. In this region, bees fall into two geographical colour forms, where the Rocky Mountain range is inhabited by species with a striking red mid‐abdominal colour pattern, and the Pacific Coastal area hosts species with a predominantly black mid‐abdomen (Ezray et al. 2019; Hines et al. 2025, Figure 1). Approximately seven *Bombus* species occur across both regions and thus switch their mid‐abdominal colour to match their regional mimicry complexes. Previous genome‐wide association studies identified the genetic basis of this parallel colour variation in two species, *Bombus melanopygus* Nylander and *Bombus vancouverensis* Cresson. In *B. melanopygus* , the alternative colour forms segregate as a discrete, Mendelian dimorphism with the red allele dominant (Owen et al. 2010) and a transition zone in Southern Oregon (Ezray et al. 2019), while co‐mimic *B. vancouverensis* displays a continuous gradient of black‐to‐red colour forms starting in the eastern Cascades and extending to the Colorado plateau. Both species were found to target the same cis‐regulatory locus of *Abdominal‐B* (*Abd‐B*); however, each used a different, independent mutation to generate this same effect. An association block with several fixed variants including a 75 bp fixed deletion was found for *B. melanopygus* , while nested within this deletion, *B. vancouverensis* has an independent 7 bp deletion that is highly associated with the colour phenotypes but is not fixed. Both deletions are associated with derived black forms from ancestral red forms (Tian et al. 2019; Hines et al. 2025).

**FIGURE 1:**
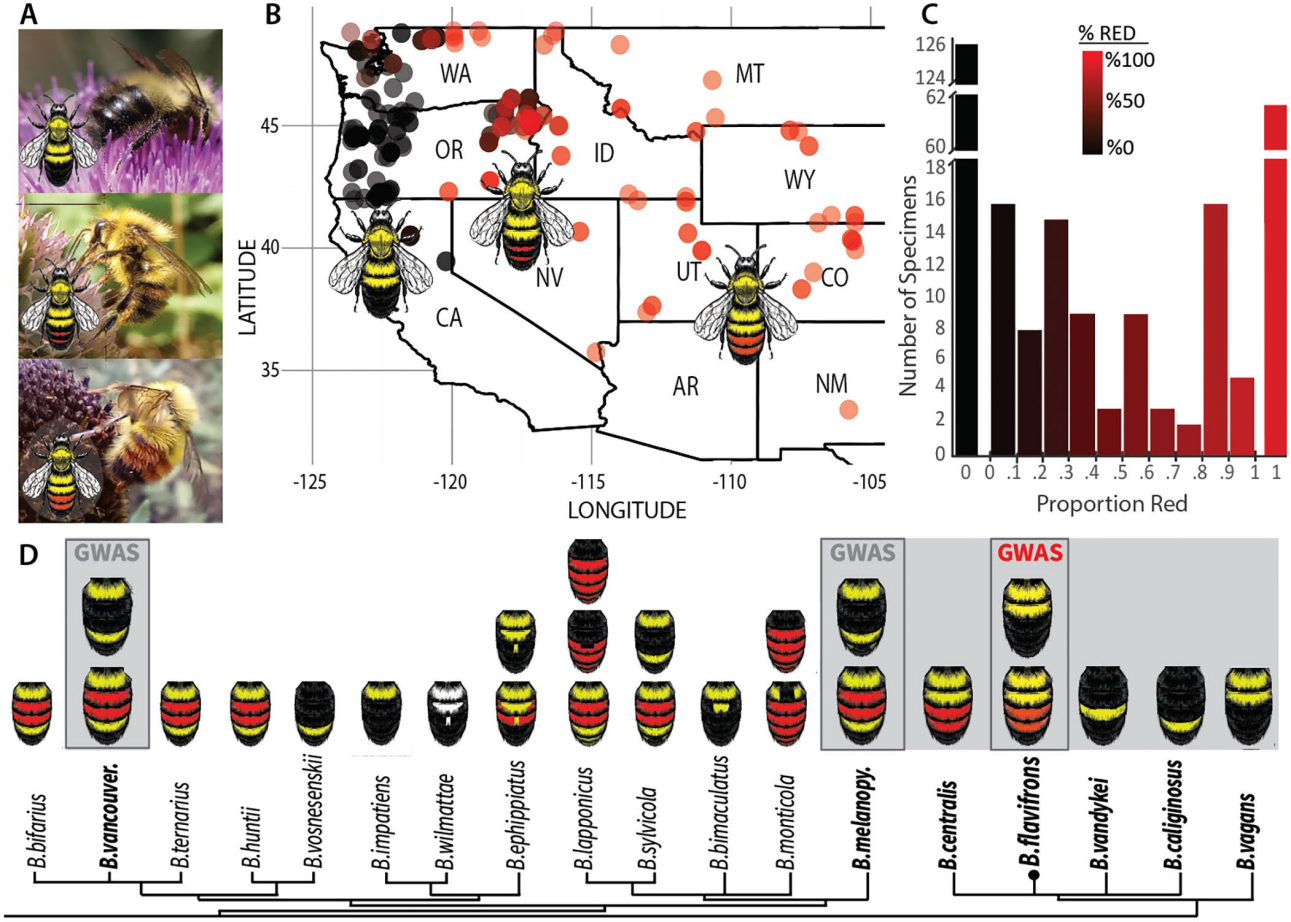
The evolution and distribution of colour forms in *Bombus flavifrons* . (A) Photos and diagrams of black, intermediate and red colour forms. Photo credits: Top—iNaturalist CC0 from selwell. Mid and bottom—T. Dabak, H. Hines. (B) The distribution of colour patterns across North America with intermediacy in mid‐abdominal coloration indicated with red‐black colour gradations. Modified from Ezray et al. (2019). (C) Histogram showing the frequency of percent red in mid‐abdominal (T3 + 4) segments across bees sampled for assessing phenotypes in (B). As the vast majority of samples are red or black, breaks are used to enable a closer look at the distribution of intermediate forms. (D) The phylogeny of North American *Pyrobombus* with colour forms of the metasoma (abdomen) indicated. Grey boxes indicate taxa studied here or studied previously. Species examined previously for GWAS (grey ‘GWAS’; Tian et al. 2019; Hines et al. 2025) or here (red ‘GWAS’) are indicated. This phylogeny is adapted from Cameron et al. (2007). *B. melanopy.* = *B. melanopygus*; *B. vancouver.* = *B. vancouverensis.*

*Abd‐B* is a member of the Hox family of homeotic transcription factors, ancient regulators that establish segmental identity along the anterior–posterior axis in insects (Hajirnis and Mishra 2021; Chen et al. 2026), and specifically is responsible for generating phenotypes in the most posterior segments. In *B. melanopygus* and *B. vancouverensis* , gene expression data demonstrate that *Abd‐B* is ectopically upregulated in more anterior segments in red forms, where it triggers upregulation of the melanin gene *ebony* which leads to the production of red pheomelanin instead of black eumelanin pigments (Tian et al. 2019; Hines et al. 2025). The black forms with deletions have reduced *Abd‐B* gene expression, to the low levels expected for these segments (Gibert et al. 2007; Singh and Mishra 2014; Tian et al. 2019). The most posterior segments (the ‘tail’) are the most common location for red colour across bumble bees and *Abd‐B* gene expression has been found to be higher in bumble bees with red tails ( *Bombus breviceps* Smith, Yang et al. 2023). Altogether, these data lead to a model for colour diversification whereby Hox genes turn on colours in their respective segments (*Abd‐B* in the tail) but can also change their location late in development (*Abd‐B* to the anterior), when pleiotropy is reduced, to move colours spatially along the body axis (Tian et al. 2019; Hines et al. 2025).

While shared utilization of *Abd‐B* in *B. melanopygus* and *B. vancouverensis* is intriguing, there are several other species with parallel red‐black polymorphisms in this North American mimicry system. In this study, we extend upon this prior work by investigating a third co‐mimicking species, *Bombus flavifrons* Cresson. *B. flavifrons* display red coloration in the Rocky Mountains and black coloration in the Pacific Coastal region, showing continuous and gradual variation from a fully red form in Utah to a fully black form in the Cascades, with maximum transitional diversity in northeastern Oregon (Figure 1). A closely related species *Bombus centralis* exhibits a monomorphic red form occurring across the Rocky Mountain region, but other closely related species are black in these segments. While the general colour patterns are similar and thus part of the regional mimicry patterns (Ezray et al. 2019), the phenotype is shifted one segment towards the tail in *B. flavifrons* compared to *B. melanopygus* and *B. vancouverensis* (Figure 1). The lineage including *B. flavifrons* also diverged earlier than the common ancestor of the *B. melanopygus* and *B. vancouverensis* clade, thus can shed light on the ancestral genetic architecture underlying mimicry across their *Pyrobombus* Dalla Torre subgenus. Prior work has shown that the 75 bp deletion region of *B. melanopygus* and *B. vancouverensis* is not variable between red and black *B. flavifrons* (Hines et al. 2025), suggesting that this lineage is utilizing another mechanism to achieve the same phenotype.

In this study, we utilize a genome‐wide association analysis to find the associated variants of the colour polymorphism in *B. flavifrons* and compare the implicated genomic region with those previously identified in comimics. We track the uncovered variants across populations of *B. flavifrons* and relative to sister lineages, considering these with respect to a revised phylogenetic framework, to better understand the microevolutionary processes by which these colour phenotypes have evolved. Ultimately, genetic and developmental research in this highly convergent system expands our understanding of how the genome is targeted in evolution, the genetics of coloration and of the genetic and microevolutionary processes promoting diversity.

## 2 Results

### 2.1 Sampling and Population Structure

**FIGURE 2:**
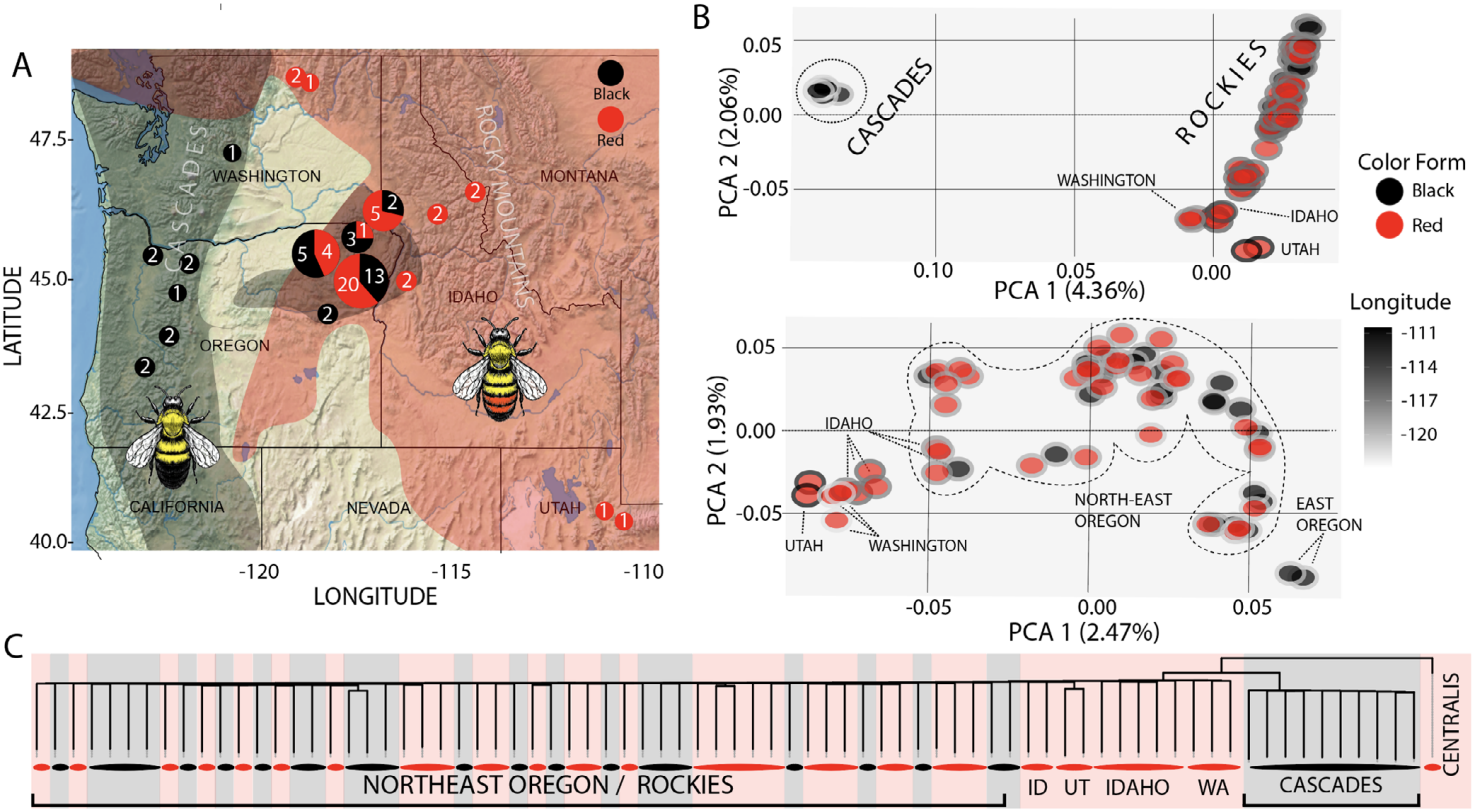
Sampling locations and their corresponding distribution areas are shown. (A) Sampling locations of bees; in the Cascade Range, only black‐form individuals occur and were collected (*n* = 10). Red forms were drawn from the distribution of fully red bees in Utah, Idaho, northeastern Oregon and northern Washington (*n* = 41) while additional black forms were sampled from the primary transition zone in northeastern Oregon (*n* = 25). At each site, a pie chart illustrates the total number of specimens and the proportion of each colour form sampled for genomic sequencing (for GWAS). To minimize overlap, adjacent or partially coincident sampling zones have been combined. (B) Principal component analysis (PCA) showing the population structure between *B. flavifrons* samples based on genomic data with the Cascade samples (top) and without the Cascade samples (bottom). Red and Black colour forms are indicated with the corresponding node colours, while the outline of PCA nodes shows the longitude relationship between the sample clusters. (C) The neighbour‐joining phylogeny inferred from genomic data across samples shows the relationships by location. State abbreviations are used as follows; ID, Idaho; OR, Oregon; UT, Utah; WA, Washington. The distribution areas in (A) are drawn using Ezray et al. (2019) and observation data from iNaturalist website (https://www.inaturalist.org/taxa/119308‐Bombus‐flavifrons). Map layers were made with Natural Earth (http://naturalearthdata.com/) https://www.naturalearthdata.com/downloads/10m‐raster‐data/10m‐cross‐blend‐hypso/.

To sample individuals for population assessment and genome‐wide association analyses, we performed whole genome sequencing on red and black form males of each colour form from across their broad transition zone, comprising 76 individuals: 35 black forms [0% red in the third and fourth metasomal tergites (T3 and T4)] and 41 red forms (≥ 90% red in these segments). This sampling included the Cascades of Oregon and Washington where there are only black forms (*n* = 10 black form; hereafter referred to as ‘Cascades black form’), the eastern Rocky Mountains where there are only red forms which include sites in Utah, eastern Washington and eastern Idaho (*n* = 11 red form), and the transitional region including parts of western Idaho and NE Oregon (30 red, 25 black) (both populations hereafter referred to as ‘eastern’ red or black forms; Figure 2A). In addition to *B. flavifrons* samples, we sequenced one outgroup specimen, *B. centralis* . Male bumble bees are haploid, thus simplifying both alignment and variant calling, and removing dominance effects for GWAS.

A principal component analysis (PCA) of genome‐wide SNP data of *B. flavifrons* specimens revealed that sample clustering was driven primarily by geographic position. Specifically, we found that the Cascades black specimens form a distinct population cluster separate from the remaining *B. flavifrons* specimens along PC1. This pattern of clustering provides strong evidence for population structure that might affect colour genetics (Figure 2B). Because this differentiation could potentially confound genotype–phenotype association analyses, we performed GWAS on sample sets both with and without the Cascades specimens. Outside the Cascades population, the colour forms were largely admixed, thus population structure is unlikely to have a strong influence on results when comparing within eastern *B. flavifrons* populations (Figure 2B). However, as their relationships follow a rough geographic gradient from Utah to Oregon and there is some sorting by colour (Figure 2B,C), we also report association analysis with population structure correction below.

### 2.2 Locus Identification Using Genomic Association

**FIGURE 3:**
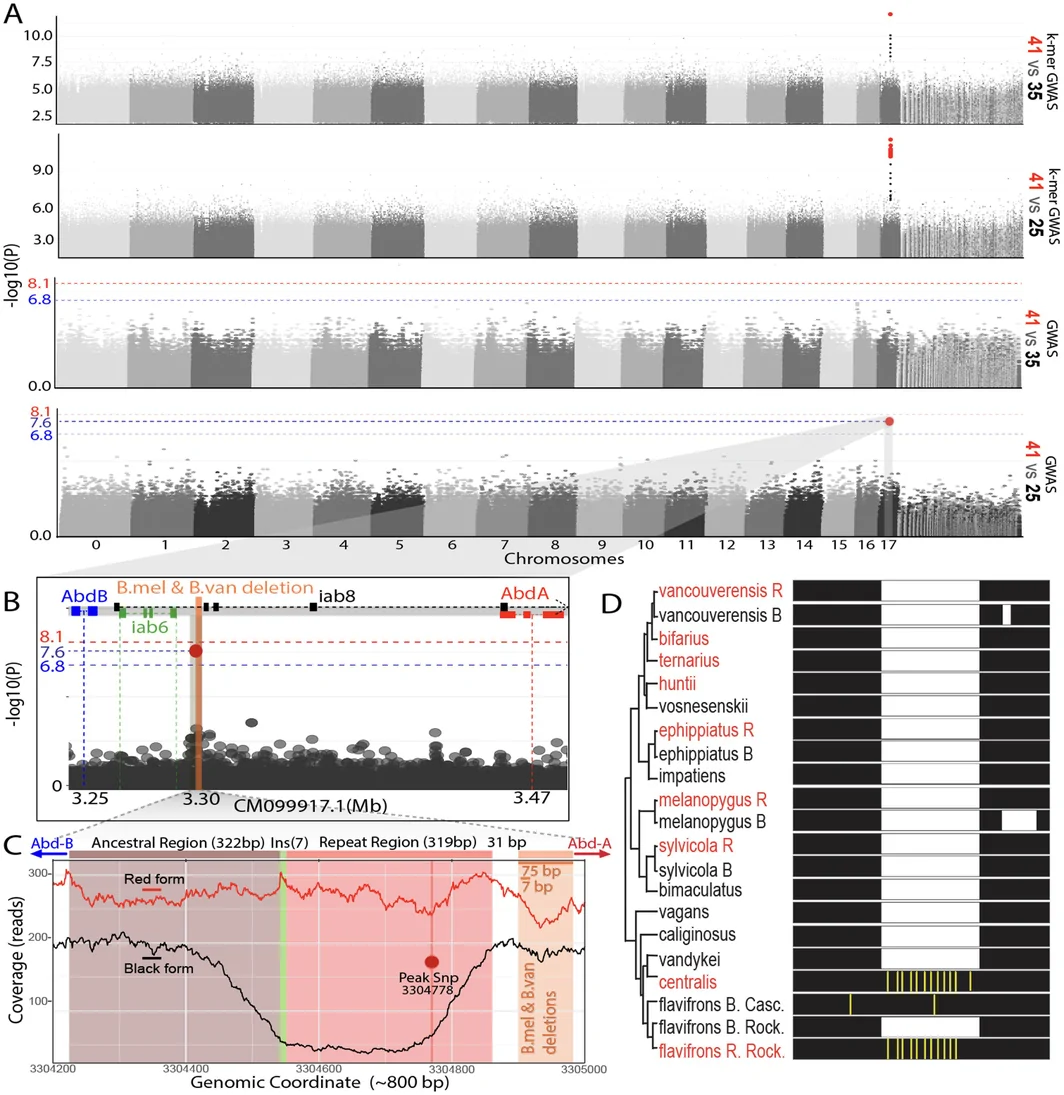
Genomic and phylogenetic context of the identified colour locus. (A) Manhattan plots spanning the genome including Cascade samples (*n* = 41 red vs. *n* = 35 black forms) and after removing Cascade samples (*n* = 41 red vs. *n* = 25 black), including separate plots for variant based and k‐mer‐based GWAS. (B) A zoomed view of the SNP region with nearby gene annotations indicated at the top. The location of the deletion region for *Bombus melanopygus* and *Bombus vancouverensis* is indicated with an orange bar. (C) Total read coverage per phenotype within the associated region, highlighting a 7 bp insertion (Ins, green area), Ancestral (brown area) and Repeat Regions (red area). The location of the *B. melanopygus* and *B. vancouverensis* deletion region is highlighted in orange. (D) Phylogenetic distribution of indel events (white bars indicate no sequence) at this locus across the North American *Pyrobombus* clade. Yellow lines indicate SNP variants that are derived from the inferred ancestral sequence. Species are coloured by their abdominal colour forms.

Genome‐wide association analysis of black and red form *B. flavifrons* haploid males did not identify any SNP or indel variants exceeding strict (hard) significance thresholds with either association analysis set (with and without Cascades samples); however, the dataset excluding the Cascades population did point to a single variant (a SNP) that rose above the background and exceeded our soft threshold (Figure 3A). This SNP mapped within the known *Abd‐B* cis‐regulatory colour locus that governs red‐black patterning in *B. melanopygus* and *B. vancouverensis* (Tian et al. 2019; Hines et al. 2025) (Figure 3B) and was not fixed between red and black forms of *B. flavifrons* .

Although standard structural‐variant callers failed to detect it, manual inspection in integrative genomics viewer (IGV; Robinson et al. 2017) led to the detection of a region with variable coverage next to the associated SNP, with red form individuals having even coverage and black form individuals having very low coverage across this region. Inspection of the sequence revealed that the red *B. flavifrons* used for the reference genome (GCA_040668555.2; Lozier et al. 2026) had a 319 bp tandem duplication at this locus, and all 41 red form bees in this study had a sequence that matched this insertion (Figure 3C). Most black individuals from the eastern population dropped in coverage in this region, suggesting they lacked this insertion, with 18 of 25 individuals lacking this insertion. The Cascades black form specimens, however, had coverage in this region (*n* = 10). The length of the duplication likely prevented its detection by short‐read structural variant calling algorithms and running the algorithms with less stringent penalties did not help with its detection. One of these tandem copies is more similar in sequence across all sequenced individuals and to outgroup taxa sequenced below, and thus we deem this the ‘ancestral’ region, while the more derived sequence is referred to as the ‘repeat’ region.

The newly detected *B. flavifrons* duplication is 31 bp away, in the direction towards *Abd‐B*, from the deletion region present in *B. melanopygus* and *B. vancouverensis* , and is within the larger 5 KB region of fixed association for *B. melanopygus* . *B. melanopygus* has two SNPs fixed by phenotype in the ancestral *B. flavifrons* region that could potentially play a role in colour pattern, but these are not variants shared with *B. flavifrons* . *B. vancouverensis* has no colour‐associated variants in this region. The originally detected SNP in *B. flavifrons* was determined to be based on false alignment of reads from the specimens of black forms lacking the duplication to the repeat portion (Figure 3C).

### 2.3 Structural Variant Analysis

We performed an alignment‐free k‐mer‐based genome‐wide association analysis to assess whether reference bias or structural or highly modified sequence variation influenced our SNP‐based association results, and to potentially find alternative loci (Willink et al. 2024; Voichek and Weigel 2020). The k‐mer‐based GWAS (Figure 3A) independently identified the same *Abd‐B* genomic region being implicated in this phenotype, this time significantly in both datasets (family‐wise error rate at 5%, corresponding to −log_10_(*p*_wald_) thresholds of 1.336 and 1.362, including and excluding the Cascade samples respectively).

A total of 45 and 35 k‐mers exceeded the significance threshold in the analyses including and excluding Cascade samples, respectively. These k‐mers showed consistent effect sizes (*β*≈−0.33) and allele frequencies (0.41–0.54 across datasets) and displayed highly correlated presence–absence patterns across individuals, indicating that they represent a single underlying sequence variant. Alignment of these k‐mers to our genome references revealed they all align to the previously identified duplication region of *Abd‐B* (Figure S1).

No additional genomic regions showed significant enrichment of associated k‐mers, thus the alignment‐free analysis did not identify a secondary candidate locus. In manually examining the 200 kb region around the implicated region across our assembled genomes, we did not detect any highly associated structural variants or SNPs other than those in the associated duplication region. Linkage disequilibrium (LD) analysis showed very low linkage across the *Abd‐B* region, with the only region of high linkage involving the linked SNPs within the duplicated region (Figure S2). We also investigated whether there were any peaks of association in the core melanin genes previously found to vary by colour forms (*ebony*, *nubbin*, *pale*, *tan*, *DDC* and *aaNAT*), but did not detect any evidence of association.

### 2.4 Insertion Characterization

To better assess the structural variant and examine the evolution of the identified locus we PCR amplified and sequenced a ~890 bp region spanning the associated tandem duplication plus the neighbouring *B. melanopygus* and *B. vancouverensis* indel region for several specimens including: (i) GWAS samples (all 76 amplified to determine insertion presence; 53 samples sequenced), (ii) intermediate male colour forms (*n* = 19 sequenced; 20 amplified; 25%–90% red), (iii) female (worker) samples to determine dominance effects of the indel variant (diploids; *n* = 21 (7 black, 8 red, 6 intermediate; all sequenced)), (iv) outgroup samples ( *B. vagans* [1 male], *B. vandykei* [1 male], *B. caliginosus* [1 male] and *B. centralis* [2 males, 1 worker]) and (v) 41 additional black form individuals from northeastern Oregon populations to increase sample sizes (41 amplified; 8 sequenced, with a focus on those with insertions).

**FIGURE 4:**
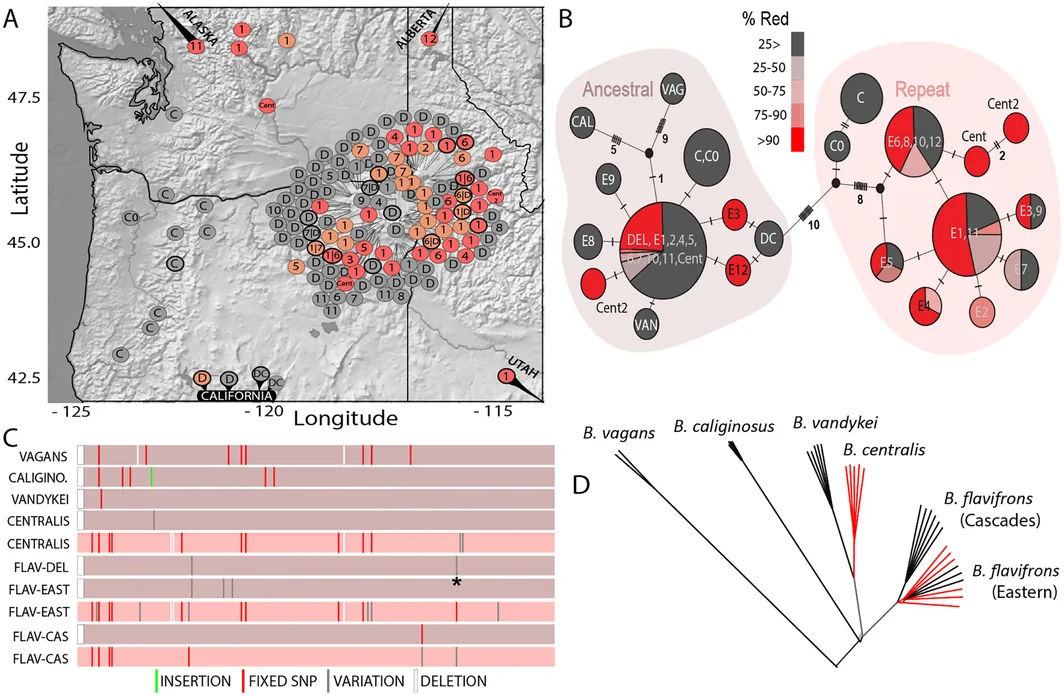
Haplotype distribution and relationships of *Bombus flavifrons* and close relatives. (A) Distribution in the NW US of different haplotypes (includes Ancestral, Repeat and the flanking *Bombus melanopygus* locus) of the colour locus. Thick outlined circles represent worker (diploid) bees, red and grey coloured circles represent red and black coloured bees whereas the orange‐brown colour represents intermediate bees. Each label inside the circles represents different haplotypes (see Figure S3 for haplotype details). Heterozygous bees are separated with the pipe (‘**|**’) symbol to show each haplotype it carries. (B) Haplotype Network of the Ancestral and Repeat region sequences. Coloration of the nodes represents the distribution of colours of a given haplotype across sampled individuals and the size of each node is representative of the sample size. (C) Colour locus alignment of Ancestral (grey) vs. Repeat (pink) regions for the most common haplotypes and for neighbouring clades. Capitalized names represent short names for the species names (e.g., FLAV = *B. flavifrons* ) and haplotypes are given as CAS = Cascades haplotype, EAST = Eastern haplotype, DEL = Deletion (non‐duplicated) haplotype. The SNP leading to the significant GWAS is shown as an asterisk (*). (D) Splitstree network showing the evolutionary relationships between *B. flavifrons* and its closest relatives based on whole genome sequence data. Branches are coloured based on the abdominal colour of the specimen. All between species and crown species nodes have 100% support (see Figure S4 for more detail). Map layers were made with Natural Earth (http://naturalearthdata.com/) https://www.naturalearthdata.com/downloads/10m‐raster‐data/10m‐gray‐earth/.

PCR amplifications and Sanger sequences confirmed that there is a tandem insertion 319–321 bp in length in this region that is very similar but not identical to the ancestral sequence and that these are linked by a 7 bp interspacer region with a unique sequence not matching the other copy (Figure 4C). Amplification of our original GWAS samples confirmed the duplicated insertion is present in all red‐form *B. flavifrons* males (41/41) and in 17/35 black form males, with all Cascades samples (black form *n* = 10) and 7 of 25 eastern black form samples having the duplication. With the indel calls correctly made, the GWAS results would have had a significant association for this duplicated insertion passing the strict threshold, with a *p*‐value of 3.62 × 10^−11^ and 7.01 × 10^−11^ when including and excluding the Cascade samples, respectively.

Population structure regression techniques, which required a LRT regression‐based GWAS instead of a Fisher's exact test, did not find the associated SNP significant with or without population structure correction. However, when we include the indel in the population correction analysis, the indel is significantly associated both with and without Cascades and with and without population correction (Figure S3).

Amplification of additional black form males found that 7 of 41 samples have the insertion; in total, all 41/41 red but only 14/66 eastern black form males have the insertion. Thus, there were three primary haplotype classes with two phenotypes that differ in both structural state and sequence: (1) an eastern insertion/duplication (red and black form), (2) an eastern without the duplication (black phenotype) and (3) a Cascade duplication (black form).

There is additional sequence diversity within these main phenotype‐haplotype classes. The red‐phenotype is the most diverse, especially in the duplication sequence, with nine distinct haplotypes. Aside from the 7 bp interspacer region, red forms average 15 bp differences between the ancestral sequence and the inserted sequence (Figure 4C, Figure S4). Some of these haplotypes are shared with intermediate‐coloured and black form individuals. Certain haplotypes are more likely to occur in intermediate or black forms (Figure 4A,B, Figure S4). Among sampled workers, homozygotes lacking the duplication are black (one diploid worker from California is intermediate), individuals heterozygous for the Eastern haplotype with and without the duplication are either black (those with haplotype 7) or intermediate in colour, and individuals homozygous for the Eastern duplication are fully red (Figure 4A,B, Figure S4).

Samples from the Cascade black form diverge into two closely related haplotypes separated by two SNPs (Figure S4). The repeat region of these haplotypes differs by only two SNPs from a reconstructed ancestral duplication, thus the duplicated sequence is more similar to the ancestral sequence than red form variants. The duplicated sequence of the Cascades haplotypes, however, also has the 7 bp interspacer and the duplicated piece is the same size as the ancestral sequence (321 bp; the red form has a few small deletions compared to the ancestral sequence), suggesting the same duplication event is likely responsible for both the Cascades and red form repeat, but that the Cascades population has a more ancestral haplotype to the more modified red form haplotypes. Eastern black form individuals without the duplication contain two distinct haplotypes varying only by two SNPs (Figure S4, Figure 4B).

### 2.5 Insertion Evolution

Relationships among species in the *Bombus* clade containing *B. flavifrons* were unresolved in prior phylogenetic analysis (Cameron et al. 2007), possibly due to rapid speciation events early in the divergence of *B. flavifrons* , *B. centralis* , *B. caliginosus* and *B. vandykei* . To improve our understanding of the history underlying polymorphisms discovered in this study, we reconstructed species and population relationships with whole genome resequencing data from female diploid workers from *B. flavifrons* (red and black eastern and Cascade black forms), *B. centralis* , *B caliginosus*, *B. vandykei* and *B. vagans* (Table S2). The genome‐wide data support the monomorphic red phenotype *B. centralis* as sister to the black‐form *B. vandykei* rather than the more phenotypically similar *B. flavifrons* (Figure 4D). This *B. centralis* + *B. vandykei* clade diverged from *B. flavifrons* ~2.3 mya, with *B. flavifrons* subpopulations (Cascades and others) diverging in the last half a million years (Figure S5). *B. caliginosus* was sister to the remaining ingroup species, with *B. vagans* as the outgroup.

The relationships among species for the insertion region (Figure 4B,C) do not match their phylogenetic history based on genome‐wide sequences (Figure 4D). At the colour locus *B. centralis* carries a fixed insertion nearly identical to the most common eastern red *B. flavifrons* haplotype (Eastern_1) (~2 SNPs difference) (Figure 4B,C). The other most closely related species (all uniformly black phenotype *B. caliginosus* , *B. vandykei* , *B. vagans* ) lacked the duplication, indicating the duplication is restricted to *B. centralis* and *B. flavifrons* (Figures 3D and 4B,C). Expanding out to red‐black polymorphic taxa in other *Pyrobombus* clades, neither the red nor the black *B. flavifrons* samples in the present dataset carry the nearby deletions in *B. melanopygus* and *B. vancouverensis* characterized in prior studies (Figure 3D; Tian et al. 2019; Hines et al. 2025) and the duplication present here is not found in other taxa, indicating this is a distinct structural event.

A haplotype network for the colour locus that separates ancestral and repeat sequence regions and aligns them together (Figure 4B) shows that outgroups root with the ancestral haplotypes, the ancestral *B. flavifrons* haplotypes cluster together with little variation, and *B. vandykei* and *B. centralis* are very similar to *B. flavifrons* in this sequence. Within the haplotypes representing the repeat region, Cascade haplotypes cluster closely and occupy a central position in the network, consistent with representing the original duplication from which other repeat haplotypes are most likely diverged. It holds a distinct position from the remaining repeat haplotypes, where *B. centralis* repeat haplotypes are similar to and seemingly derived from *B. flavifrons* repeat haplotypes. The *B. centralis* specimens sequenced do not harbour the sequence diversity found in *B. flavifrons* . Individuals that have repeat haplotypes that are black or intermediate in colour are distributed across the repeat haplotype network.

To examine whether historical introgression or incomplete lineage sorting might explain the distribution of the repeat alleles within *B. flavifrons* and closely related species, we tested for excess allele sharing using Patterson's *D* statistic (i.e., the ABBA‐BABA method) and *f4*‐ratio admixture fractions. We compared the frequency of sharing of alleles between either Cascades *B. flavifrons* or eastern *B. flavifrons* populations and either member of the *B. flavifrons* sister lineage— *B. centralis* (red form) + *B. vandykei* (black form). We found evidence for significant excess allele sharing for *B. centralis* with both *B. flavifrons* populations and for *B. vandykei* with the Cascades *B. flavifrons* population (Figure S6). Tests for spatial clustering of informative SNPs within the genome, which may be indicative of true introgression versus scattered single‐SNP values, were significant in all comparisons for the sensitive statistical test but not for the more stringent robust test of clustering (Figure S6), providing some evidence for introgression but not definitively ruling out other processes like homoplasy. Examining *f4*‐ratios across the phylogeny using the *f‐branch* test shows a general pattern where *B. centralis* shares more derived alleles with both *B. flavifrons* lineages than *B. vandykei* , and *B. vandykei* shares more alleles with the Cascades *B. flavifrons* than the eastern *B. flavifrons* population (Figure S6). We interrogated the colour locus for patterns of introgression, but windows overlapping the locus did not have detectably larger or smaller *D* values than other genomic intervals.

### 2.6 Transcription Factor Binding Site and Enrichment Analysis

**FIGURE 5:**
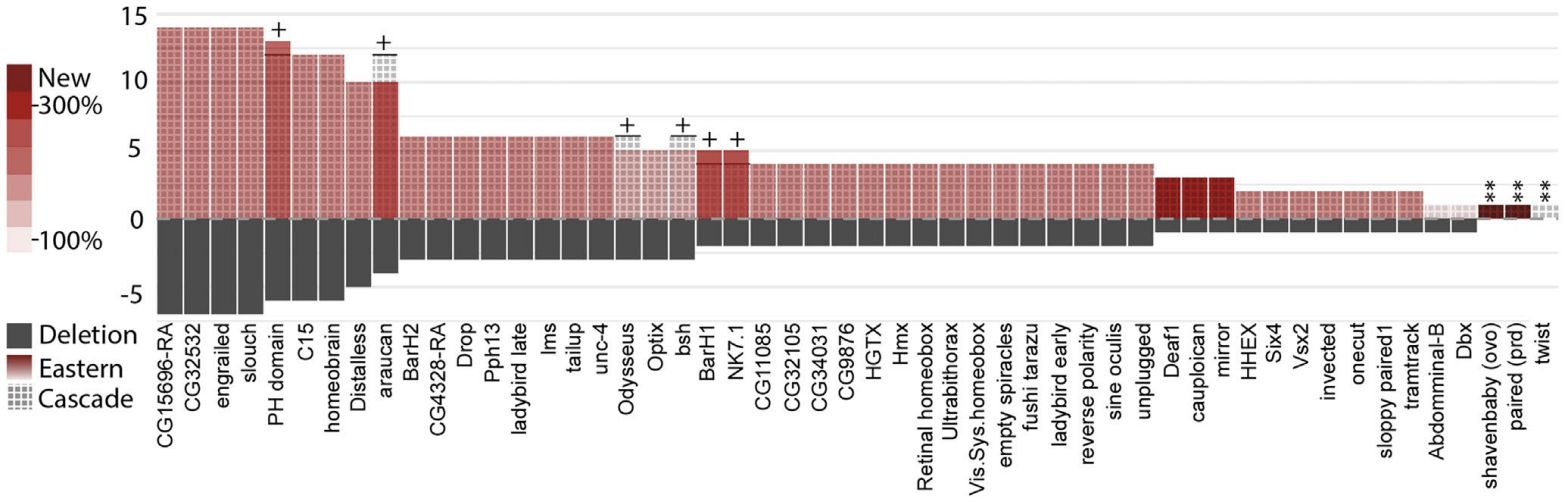
The number of predicted transcription factor binding sites (TFBS) for the deletion (black) vs the Eastern (mostly red) and Cascades (dotted) haplotypes. Plus (+) sign indicates the TFBSs that show difference between the Eastern and the Cascades haplotypes whereas the vertical asterisks (⁑) show the new TFBSs introduced in haplotypes that contain the duplication.

Transcription factor binding site (TFBS) prediction was performed on a total of 20 Sanger‐sequenced haplotypes varying across both the Repeat and the Ancestral region of the indel (Figure S7). The haplotype of eastern bees without the duplication contains 134 predicted TFBS whereas the most abundant haplotype of the eastern bees with the duplication has 273 sites, with duplication haplotypes ranging from 272 to 275 sites for each sample; thus, the duplication more than doubles the total number of predicted binding sites. The number of motif genes, however, is similar (49 vs. 51) with only *shavenbaby* and *paired* unique to the duplicated sequences. Motifs that show the most differences in count number with the insertion are *Deaf1*, *caupolican*, *mirror*, *BarH1*, *NK7.1*, *araucan* and *PH domain* TFs (Figure 5). The enrichment analysis containing these 9 motifs against the 143 background TFs revealed that the duplication‐driven haplotypes were enriched for muscle cell fate commitment, equator specification and negative regulation of growth (Figure S8).

The Cascade haplotypes (black form) show motif counts very similar to the Eastern insertion haplotypes (black and red forms), with only slight gains or decreases in some transcription factors and no cases where the count number is as low as the eastern haplotypes without the duplication (black form) (Figure 5). Thus, the additional modifications in the red forms do not have obvious TF‐binding effects. Within the eastern populations, haplotypes associated with black and intermediate phenotypes show only a few deviations in predicted TFBS counts relative to the most common eastern (Eastern_1; mostly red) haplotypes (Figures S4 and S7). Some insertion haplotypes belong to more black and intermediate compared to red individuals, such as Eastern_7, 10 and 11 (and 8 and 9 singletons) (Figure 4B, Figure S4); however, there is no obvious common TF change with these, aside from some having a reduction of *araucan* binding, a TF site with one of the highest increases in representation in the red form and which is the most likely to change across haplotypes (Figure 5, Figure S7).

## 3 Discussion

### 3.1 Convergent Hox‐Regulatory Evolution Across Mimics

This study provides evidence for genomic hotspots playing a role in the evolution of colour variation in bumble bees. Our study of the parallel red‐black colour polymorphisms in a third co‐mimicking bumble bee species spanning the western North American mimicry zones, *Bombus flavifrons* , revealed that the species utilizes the same 500 bp cis‐regulatory locus implicated in red and black dorsal pigmentation in co‐mimics *B. melanopygus* and *B. vancouverensis* , but that mutations in each of these three species have arisen independently. In *B. melanopygus* and *B. vancouverensis* , the black phenotype is associated with small deletions (75 bp for black form *B. melanopygus* and 7 bp for black form *B. vancouverensis* ) in this *Abd‐B* cis‐regulatory region, whereas in *B. flavifrons* we recover a nearby tandem duplication (319 bp) and a novel insertion (7 bp) that is associated with red coloration (Tian et al. 2019; Hines et al. 2025). That independent mutations involve the same cis‐regulatory interval implicates altered *Abd‐B* regulation, specifically in this region, as the optimal way to generate colour change across these species and points to the power of mutation to generate similar phenotypes by targeting the same hotspots for change, even among close relatives.

Although we do not have gene expression data for *B. flavifrons* due to challenges in rearing this species, prior studies have found that *Abd‐B* gene expression creates black‐red shifts in its co‐mimics by *Abd‐B* altering melanin genes such as *ebony* (Tian et al. 2019; Hines et al. 2025). Even a much more distantly related species, *B. breviceps* (subgenus *Alpigenobombus*), has mutations that modify the expression of *Abd‐B* for red and black abdominal variation (Yang et al. 2023). Based on our sequencing evidence, we thus hypothesize that *B. flavifrons* is using the same mechanism involving a shift in *Abd‐B* expression to shift coloration from black to red.

Given patterns in *Drosophila* (Kronforst et al. 2012; Massey and Wittkopp 2016), one would predict colour patterns in bumble bees would be generated by targeting downstream pigmentation genes in cis‐ to alter their interaction with segmental Hox proteins, as modifying Hox gene expression is known to generate highly pleiotropic homeotic transformations. Yet, for all three bumble bee species in this mimicry complex, modification of *Abd‐B* expression using the same regulatory locus is implicated. Modularity in the *Abd‐B* regulatory region controlling its tissue and time specific expression combined with the ability of this gene to create segment‐specific effects, may explain why it is the optimal means to acquiring these segmental colour patterns. In *Heliconius*, pleiotropic developmental genes are also targeted, where they are co‐opted in a new context for wing development. These too are hotspots, as multiple species find the same solution by targeting mutations in the same larger regulatory region of the same genes (Supple et al. 2013; Martin et al. 2012; Gallant et al. 2014). Together these results show that upstream developmental genes are good targets when utilized for context‐specific expression that avoids pleiotropy.

### 3.2 Evolutionary Genetics of a Hox Hotspot in *B. flavifrons*

The pattern we observe is consistent with an evolutionary model in which a tandem duplication provides a space for adaptive innovation. In this scenario, duplication of the *Abd‐B* cis‐regulatory element preserves the ancestral regulatory function in one copy (ancestral) while allowing the second copy (repeat region) to potentially accumulate mutations and novel transcription factor binding sites that can give rise to a derived regulatory mechanism. Predicted TF‐binding analyses suggest that duplication‐carrying haplotypes most likely amplify motif number relative to the ancestral haplotypes lacking the duplication, however, there was a gain of new motifs for *shavenbaby* and *paired* with this duplication. *Shavenbaby* controls setal development (Chanut‐Delalande et al. 2006), which is where the colour is located; thus, this may impact setal‐specific upregulation of *Abd‐B*. These new binding sites came about from the novel 7 bp interspacer between these sequence copies, suggesting this could be an important element of this duplication process. Araucan TF‐binding also showed considerable variation between morphs and among haplotypes. This gene regulates sensory bristles in *Drosophila* via *achaete‐scute* (Simpson 1996; Ikmi et al. 2008) and is responsible for structure of scales in butterflies (Chatterjee et al. 2025), thus, it could be involved in homologous setal regulation in these bees.

Changing binding site frequency can alter threshold responses to genes, allowing downstream genes like *Abd‐B* to potentially respond to lower levels of upstream proteins. A similar pattern has been reported in *Bombus niveatus* , where a duplication in the cis‐regulatory region of *BarH* is thought to shift TF‐binding frequency to generate a yellow‐white mimetic colour switch (Dabak et al. 2026). Structural variants like regulatory duplications are increasingly recognized as an important source of genetic variation contributing to adaptive phenotypes (Stuart et al. 2026). Where better characterized in *Drosophila*, indels were noted to comprise ~16% of all variants and drive 10% of expression quantitative trait loci, having a larger effect on phenotype than SNP variants (Massouras et al. 2012). Although most studies continue to focus on SNPs, such results suggest that a greater emphasis on small structural variants will likely be important for understanding environmental adaptation.

The segmental position of the abdominal tergites experiencing red‐black dimorphism in *B. flavifrons* is shifted one segment posterior (T3–T4) relative to the pattern in co‐mimics *B. melanopygus* and *B. vancouverensis* (T2–T3). In *Drosophila*, the regulatory region between *Abd‐A* and *Abd‐B* operates in a colinear fashion (Celniker et al. 1990; Graveley et al. 2011; Garaulet and Lai 2015) and thus regions closer to *Abd‐B* will regulate expression of *Abd‐B* in more posterior segments. In *B. breviceps* the colour locus is closer to *Abd‐B* and in this case the more posterior tail colour is modified, thus following this model. In *B. flavifrons* , the implicated domain has only a slight 31 bp shift towards *Abd‐B*. The implicated locus falls in an intron of a long‐non coding RNA (*iab‐8* lncRNA) that spans the abdominal Hox genes, falling near an alternatively spliced terminal exon that has similarities to a similar alternatively spliced lncRNA (iab msc) terminal exon in *Drosophila* (Hines et al. 2025; Graveley et al. 2011), thus this region could have an important conserved function. The posterior‐shifted colour pattern could also be impacted by an interaction with genes controlling yellow colour, as these species also vary in which segments are programmed to be yellow (T4 in *B. melanopygus* and *B. vancouverensis* ; T2 in *B. flavifrons* ), a switch that happens earlier in development than melanic regulation.

The contrast between the dimorphic *B. melanopygus* and the polymorphic species ( *B. vancouverensis* and *B. flavifrons* ) suggests that the genetic architecture of black coloration is variably impacted by other loci. In the dimorphic *B. melanopygus* , the Hox locus alone likely drives the red‐black switch as it was the only associated locus, had variation fixed by phenotype, and prior crossing studies determined this dimorphism was driven by two alleles at a single locus in a Mendelian fashion. In contrast, both *B. vancouverensis* and *B. flavifrons* show continuous variation in the amount of red/black pigmentation and the implicated Hox locus is not fixed by colour. In *B. flavifrons* , all red individuals are fixed for the insertion, suggesting its important role in red colour; however, a few of the northeast Oregon black forms have the same insertion as the red forms, and the Cascades black form bees also have an insertion but with a more ancestral sequence. Similarly, in *B. vancouverensis* , all red form individuals were fixed in not having the implicated deletion, but some black form individuals also lacked the deletion (Hines et al. 2025). The partial sorting of intermediate and black phenotypes by haplotypes at the colour locus may suggest that the continuous variation at this locus may be impacted by a complex combination of variants around this locus, as has been found in *Drosophila* coloration (Rebeiz et al. 2009, 2015). To create this colour continuum, however, it is likely that additional loci in other pigmentation‐altering genes may lead to black coloration.

Prior transcriptomic research (Hines et al. 2025) shows that black is a default state, as producing red pheomelanin instead of black eumelanin typically involves the upregulation of *Abd‐B* in a new location, which triggers *ebony*, perhaps using the gene *nubbin* as an intermediate. The generation of black forms can occur through any mechanism that prevents *ebony* upregulation in setae, but multiple other genes in the melanin pathway can also lead to altered ratios of red and black melanin (Hines et al. 2025). Our analysis showed no clear secondary location detectable with bioinformatics within the colour interval, near melanin genes, or elsewhere in the genome. Thus, there likely are several modifier loci. A lack of a clear secondary peak is not unexpected, given that if only two loci were involved the variation would be less continuous than is observed (Figure 1). Resolving alternative loci would benefit from denser sampling, especially of black individuals with the insertion, as well as functional genetics approaches.

### 3.3 Population Processes

The phylogenetic structure of the duplicated copy and the haplotypes of both the ancestral copy and the duplicated region suggests the insertion evolved from a black‐form ancestor that lacked the repeat. The Cascades black‐form population appears to represent an ancestral haplotype representing the original insertion as both copies are very similar in sequence whereas the eastern duplicated haplotypes of *B. flavifrons* have a repeat sequence that has heavily diverged from the ancestral copy. Most striking is that the red forms of *B. flavifrons* share nearly the same derived SNPs in the repeat region as *B. centralis* , a related species that is exclusively red form. *B. centralis* is most closely related to another species, *B. vandykei* , that is exclusively black form and lacks the insertion. Because this allelic distribution does not match the phylogenetic history, either alternative sorting of ancestral variation or introgression must have led to these phenotypes. In the case of introgression, the red form insertion haplotype of *B. flavifrons* could have introgressed into *B. centralis* after their split with the other species and subsequently became fixed, or the black form populations of *B. flavifrons* may have acquired the deletion through introgression with black sister lineages. Comparative examples from *Heliconius* butterflies show that some species have acquired adaptive regional mimicry phenotypes through hybridization and subsequent introgression of wing‐pattern alleles from mimicking species (Pardo‐Diaz et al. 2012; Martin et al. 2013; Edelman et al. 2019; Rossi et al. 2024).

The data support a most likely scenario where the three variants in *B. flavifrons* arose during a time prior to the divergence of the *B. flavifrons–B. centralis–B. vandykei* clades and sorted in these lineages thereafter. Our data suggest the possibility that some post‐divergence introgression occurred between *B. centralis* and *B. flavifrons* and between *B. vandykei* and black Cascades *B. flavifrons* , a geographic restriction which makes sense given *B. vandykei* only occurs in the western region. Such historic introgression is not unexpected given the rapid evolution that has confounded determination of phylogenetic relationships among species in this clade (Cameron et al. 2007; Figure S2). Nevertheless, adaptive introgression of the colour locus does not have strong support, as we find no clear signal of introgression for SNPs surrounding the colour locus that differs from the rest of the genome. Additional population level sequencing, especially of *B. centralis* , is needed to better understand the history of these alleles.

Our study also supports the potential for different populations to use different loci and mutations for the same phenotypes. Black forms from northeast Oregon show different patterns of regulation than black forms from the Cascades. Cascades bees showed a distinct population structure suggestive of a history of isolation, where strong mimetic selection for black colour forms in the Coastal region could have independently selected for different alleles both within and outside of the colour locus. Similarly, in *Drosophila*, patterns of melanism have been shown to involve different genes in different populations (Pool and Aquadro 2007; Kronforst et al. 2012; Bastide et al. 2016; Massey and Wittkopp 2016; Berardi et al. 2025). These data together suggest that while allele sorting and introgression can be important for leading to phenotypic variation, populations also can reach different adaptive solutions to the same problem; thus, it is important to not underestimate the potential of mutation.

### 3.4 Challenges with GWAS in the System

The association analysis in this study presented both biological challenges and practical limitations. A high recombination rate combined with necessary sampling of diverse populations reduced the signal to a single, albeit false, variant. Weak linkage makes it more difficult to find regions, but with the converse advantage of targeting single variants and thus being able to identify more likely functional sites. Furthermore, the likely multigenic inheritance in *B. flavifrons* presents a need to sample many bees, and full red and full black phenotypes (i.e., not intermediate in coloration) from the transition zone are rare. Sampling across a broader region, as was initially done in our study, leads to population structure that increases background signal and complicates analysis (Sillanpää 2011; Hellwege et al. 2017; Sul et al. 2018), while also potentially resulting in different alleles being implicated across different regions, as was found in this case.

From a bioinformatic perspective, the short‐read pipeline was unable to identify medium‐sized structural variants such as the tandem duplication. Thus, studies may miss structural variants if they do not employ long‐read approaches. Long‐read sequencing would allow direct discovery and phasing of structural variants and paralogous copies of any duplications, removing the reliance on depth‐based screening and manual IGV inspection for structural variant detection. The k‐mer‐based GWAS approach was able to overcome some of these limitations. This approach may thus be a valuable complement to standard GWAS when structural variation may be contributing to a trait of interest.

In other bumble bees, we have been able to enhance our understanding of gene function with investigations of gene expression of epidermal tissue during pigment development in laboratory‐reared colonies (Hines et al. 2025). Unfortunately, *B. flavifrons* is difficult to rear under laboratory conditions (personal observation; Strange et al. 2023). Despite repeated attempts over the past decade, we have not been successful in establishing laboratory colonies capable of producing the developmental stages required for gene‐expression or functional genomic experiments. Consequently, approaches such as developmental RNA sequencing, reporter assays, RNAi or CRISPR‐based validation are not currently feasible for this species at this time. Development of functional genetic techniques among these comimics will enhance our understanding of the role of this locus and gene in coloration.

## 4 Materials and Methods

### 4.1 Genome‐Wide Association Analysis

DNA was extracted from 76 males for the genome‐wide association analyses (details in Table S1A). Extractions were performed on two legs and thoracic muscles for each sample using Monarch DNA extraction and Zymo Quick‐DNA Miniprep kits with standard protocols with RNase removal. For these samples, DNA libraries were prepared with NEBNext Ultra Express FS DNA Library Prep kits followed by sequencing in two separate Illumina short‐read sequencing runs. Paired‐end read sequencings were performed on the first run using the NextSeq 2000 P3 (150 bp ~15–20× coverage) and the second run using the NextSeq 2000 XLEAP P4 (100 bp ~18–22× coverage) sequencers. Library preparation and sequencing were conducted at the Penn State Huck Genomics Core Facility (RRID:SCR_023645) (University Park, PA) (Table S1A).

Each raw sequencing file was assessed for sequencing quality using FastQC v.0.11.9 (Andrews 2010), MultiQC v.1.21 (Ewels et al. 2016) and SeqKit v.0.15.0 (Shen et al. 2016). Adapters and low quality bases and reads were removed using trimmomatic v0.39 (MINLEN:36 LEADING:3 TRAILING:3) (Bolger et al. 2014) (Quality Phred score cutoff < 30). Overall, the data were of high quality, with only 6%–9% of reads removed during trimming. Filtered reads were aligned to the *Bombus flavifrons* genome assembly (iyBomFlav1, RefSeq ID GCF_040668555.1, GenBank GCA_040668555.2) (Lozier et al. 2026), which was collected in Utah and had a red phenotype (40.72091 N 110.86758 W), using the BWA aligner v 0.7.17 (Li and Durbin 2009) on bwa‐mem setting (Li 2013) with the strict parameter (‐T 40). Read group information was added to alignment files via Picard tools v.2.23.9 (available from: https://broadinstitute.github.io/picard/index.html) and indexed using sambamba v1.0.0 (Tarasov et al. 2015). Duplicated reads were removed with GATK v.4.4.0.0 using *MarkDuplicatesSpark* function. Sequencing depth was assessed using total sequenced bases per individual after read processing, and mean read depth per sample was inferred post‐alignment. Sequencing depth was comparable between black and red morphs (Wilcoxon rank sum test [*W* = 825, *p* = 0.2787]) as was mapping depth, with median aligned depths of 12.3× (black) and 12.0× (red) and mapping rates of 99.6% for both.

Variant calls from these sample‐specific alignment files were conducted using GATK v.4.4.0.0 (McKenna et al. 2010) using the *HaplotypeCaller* option with haploidy and base specific settings (*‐‐ploidy 1 ‐ERC BP_RESOLUTION*) to generate per‐sample Genomic VCFs (GVCFs). Two sets of GVCFs were aggregated separately due to inferred population structure (Figure 2); (i) one analysis was conducted on the full dataset (76 samples with 35 black/41 red forms, 6,321,393 SNPs) and (ii) the other analysis was conducted after removing the samples (10 black forms) from the Cascades that represent a distinct population from the rest of the sample (66 samples, 25 black/41 red forms, 5,303,715 SNPs). These GVCFs were aggregated for each dataset using *GenomicsDBImport* functionality and the *GenotypeGVCFs* option was used for joint genotyping across all samples to produce a unified cohort‐wide variant calling dataset. This raw variant call file was used to identify SNPs and indels using *SelectVariants* function and these files were then filtered using *VariantFiltration* function using parameters (*QD* < *2.0, FS > 60.0, MQ < 40, SOR* > *4.0, MQRankSum < −12.5, ReadPosRankSum < −8.0*) following best practices with GATK guidelines (Van der Auwera et al. 2013; Van der Auwera and O'Connor 2020). Because no validated set of known polymorphic sites exists for *Bombus flavifrons* , these filtered high‐confidence SNP and indel files were then used to generate calibration files for the alignments via the BaseRecalibrator function and the alignments were calibrated using these files with *ApplyBQSR* function.

Finally, calibrated alignments were used for final variant calling via bcftools v1.16 (Li 2011) using *mpileup* and *call* functions for haploid samples (*‐‐ploidy 1*). We assessed population structure among samples for the full dataset and the dataset without Cascades samples. For this, each variant dataset was linkage disequilibrium (LD)‐pruned in PLINK v1.90b6.21 (Purcell et al. 2007) using the ‐‐indep‐pairwise 50 kb 5 0.1 option. This retained 260,216 and 183,655 variants for the with and without Cascade sample datasets, respectively. Pairwise genetic distances were then calculated from the LD‐pruned dataset using the parameters *‐‐vcf‐half‐call haploid ‐‐allow‐extra‐chr ‐‐allow‐no‐sex ‐‐distance‐matrix ‐‐double‐id ‐‐no‐parents*. The resulting distance matrix was imported into R v4.2.2, where classical principal component analysis was performed and results were visualized using the tidyverse v2.0.0 package (Wickham et al. 2019). A distance matrix generated of the full dataset plus the addition of a *Bombus centralis* sample, was also used to construct a neighbour‐joining phylogenetic tree using the ape R package v5.7 (Paradis et al. 2004).

The unpruned version of the filtered variant call dataset was run through a genotype–phenotype association analysis using case vs. control phenotypes (black vs. red) and a Fisher's exact test in PLINK v1.90b6.21, with Manhattan plots generated in the qqman R package v0.1.9 (Turner 2018). To further evaluate whether population structure influenced the association or masked any additional peaks, an additional genome‐wide association analysis was performed correcting for population structure using REGENIE v4.1.2 (Mbatchou et al. 2021). For this analysis we also added a binary marker representing the duplication presence or absence that was manually encoded. Binary‐trait association testing was performed using Firth penalized logistic regression with approximate likelihood‐ratio testing (LRT) (‐‐firth ‐‐approx) while including the first two principal components from above (PC1 and PC2) as covariates. Variants with a minor allele count (MAC) less than three were excluded from the analysis.

To determine significance thresholds for genome‐wide association results, we implemented a stringent Bonferroni‐corrected threshold involving a Bonferroni correction followed by dividing the threshold level (0.05) by the total number of SNPs and transforming the result to the −log_10_ scale: −log_10_(0.05/*n*), where *n* is the total number of SNPs analysed. We also identified a softer threshold of −log_10_(1/*n*) corresponding to the *p*‐value expected by chance for a single test across the genome. SNPs exceeding these thresholds were considered candidates for further annotation and downstream analyses.

### 4.2 Candidate Locus Validation

We designed primers to amplify an ~890 bp region that included the 319 bp tandem duplication, the ancestral duplicated sequence, and the indel region for *B. melanopygus* and *B. vancouverensis* . PCR primers were designed (5′‐AGGGATTGAAGAGGAAAACAGAA‐3′ and 5′‐TGTTATCGTACCCAAGTCGG‐3′ ~52.5°C annealing temperature) flanking the using Primer3web v4.1.0 (Koressaar and Remm 2007; Untergasser et al. 2012; Koressaar et al. 2018). Amplicons of this region were first visualized on gels to confirm the presence/absence of the insertion which led to the selection of a subset of the samples (*n* = 54) for Sanger sequencing (Table S1). PCR products were purified for sequencing using ExoSap and sequences were generated at the PSU Huck Genomics Sequencing Center (University Park, PA). Sequencing was performed largely from the forward primer, but in cases with heterozygosity we also sequenced the reverse strands as these generated double peaks due to the indel variant. Sequences were manually edited and aligned using Geneious v8.1.9 (available from: https://www.geneious.com) and chromatograms were double checked for all variants post‐alignment.

### 4.3 Structural Variant Analysis

To look for structural variants, we performed k‐mer GWAS using KMC v3.1.0 (Kokot et al. 2017). For this, 31‐mers were counted across all samples, filtered by minimum allele count ≥ 5 and presence in ≥ 20% of samples, and tested for association with phenotype using a linear mixed model implemented in GEMMA v0.98.5 (Zhou and Stephens 2012), incorporating a kinship matrix estimated from k‐mer presence‐absence patterns. Significance thresholds were determined by phenotype permutation as described previously (*family‐wise error rate at 5%, corresponding to −log*_*10*_ *(pwald) thresholds of 1.336 and 1.362*). Significant k‐mers were mapped back to the reference genome using BLAST v2.17.0 (Camacho et al. 2009) to identify their genomic context. After filtering for k‐mer frequency, approximately 101 million and 94 million k‐mers were retained for association testing in the dataset including and excluding the Cascade samples, respectively.

We also generated three *de novo* genome assemblies for each of the main haplotypes (black phenotype with deletion [TDB023], black phenotype with insertion [TDB010], Cascades [TDB004]) using SPAdes v4.2.0 (Prjibelski et al. 2020). We compared assembled sequences to the 200 KB region surrounding the identified associated locus to both Sanger data and the *B. flavifrons* reference genome (GCA_040668555.2) sequence to assess whether any additional variants were missed.

An LD analysis spanning window sizes of 4, 10, 20, 30, 50 and 100 kb for the associated region was conducted using PLINK v1.90b6.21 (‐‐allow‐no‐sex ‐‐double‐id ‐‐vcf‐half‐call haploid ‐‐r2 ‐‐ld‐window‐r2 0 ‐‐chr CM099917.1 ‐‐allow‐extra‐chr).

### 4.4 Transcription Factor Binding Site (TFBS) Analysis and Enrichment

The TFBS analysis was conducted using Biostrings v2.66 (Pagès et al. 2022), TFBSTools v1.36 (Tan and Lenhard 2016) and JASPAR2020 v0.99.10 (Baranasic 2020) packages in R v4.2.2. JASPAR CORE collection was utilized using JASPAR2020 to obtain insect‐specific position‐weight matrices (PWMs) and their probabilistic PWMs were calculated with TFBSTools using *Bombus*‐optimized background nucleotide frequencies (*A* = 0.31, *C* = 0.19, *G* = 0.19, *T* = 0.31) (Crowley et al. 2023). Motifs were filtered at a 90% relative‐score threshold. Motifs of each haplotype were assessed by genomic coordinates using dplyr v1.14.0 (Wickham et al. 2023), ggplot2 v3.5.1 (Wickham 2016) and tidyr v1.3.0 (Wickham et al. 2025) packages.

Resulting TFs with greater than or equal to 2 fold representation between the insertion and deletion haplotypes were used as enrichment dataset queries and the background for this enrichment analysis was generated from the full set of TFs in the JASPAR2020 CORE database. JASPAR IDs from each dataset were converted to Flybase IDs from the ensembl metazoa biomart webtool (https://metazoa.ensembl.org/biomart) and then analysed on the DAVID webserver (Huang et al. 2009; Sherman et al. 2022).

### 4.5 Haplotype Network

Sanger sequences were used to generate a haplotype network of the identified locus. For this, sequences were divided into two sequence regions (Ancestral and Repeat) to better assess the allelic origin of the duplication, with the 7 bp interspacer assigned to the Repeat sequence. Each sequence block was aligned to each other using Geneious. The aligned dataset was then used to generate a haplotype network using the TCS algorithm implemented in the Hapsolutely tool (Vences et al. 2024).

### 4.6 Species Tree Inference Using Whole Genome Sequences

To infer the phylogenetic relationships of *B. flavifrons* relative to sister lineages, we performed whole genome sequencing on 31 female diploid workers, including eastern *B. flavifrons* (red forms from Colorado and Utah, *n* = 2; red forms from the transition region, *n* = 4; black forms from the transition region, *n* = 3), Cascades *B. flavifrons* black forms (*n* = 6), *B. centralis* (red, *n* = 5), *B*. *caliginosus* (black, *n* = 4), *B. vandykei* (black, *n* = 5), and *B. vagans* as the outgroup (*n* = 2) (Table S2). DNA was extracted from thoracic muscle using the Qiagen DNeasy Blood & Tissue kit (Qiagen, Hilden, Germany) and prepared for sequencing by constructing whole genome sequencing libraries using the NEB Ultra II FS DNA Library Prep Kit (NEB, Ipswich, MA, USA) following the manufacturer's instructions and using unique dual indexing. Equimolar pooled samples were then sequenced using the NovaSeq X Plus technology (PE 150) at Psomagen Inc. (Rockville, MD, USA). Reads were trimmed using TrimGalore! (https://www.bioinformatics.babraham.ac.uk/projects/trim_galore/; Martin 2011) with default settings except quality set at *2colour = 20*, which resulted in a mean of 21.0 ± 6.2 SD million read pairs and 6.1 ± 1.7 SD Gb of data per sample (sample information in Table S2). Reads were aligned to the *B. flavifrons* reference genome and read group information was added using bwa‐mem 0.7.15‐r1140 with default settings, followed by sorting with samtools 1.10 (Danecek et al. 2021), marking duplicate reads with picard MarkDuplicates 2.23.9 (https://broadinstitute.github.io/picard/) and indexing the final BAM with picard BuildBamIndex. Variants were called using the GATK 4.6.1.0 HaplotypeCaller, GenomicsDBImport and GenotypeGVCFs to produce a final joint genotyping VCF for each reference genome scaffold. Scaffolds for the 18 identified chromosomes in the *B. flavifrons* genome were combined and the genotypes were filtered to remove insertion‐deletions and retain bi‐allelic SNPs with a minimum sequencing depth of 10, a minor allele count of 2, and < 20% missing data across samples. This produced a VCF with 4,150,897 SNPs with a mean depth of 16.7 ± 5.0 SD per sample.

Given prior difficulty in resolving relationships in this clade, we first used a splits network approach implemented in SplitsTree 6.6.2 (Huson and Bryant 2024). We converted the VCF to phylip format with vcf2phylip (Ortiz 2019) specifying one *B. vagans* as the outgroup (BRL1260_vagans) and randomly resolving IUPAC ambiguities for heterozygous positions, followed by a final filter to remove any residual invariant sites imposed by this resolution. This produced a final data set containing 3,920,277 bp. The alignment was imported into SplitsTree 6.6.2 and the neighbour‐net algorithm (Bryant and Moulton 2004; Bryant and Huson 2023) was used on p‐distances to produce a split network with 100 splits bootstraps to test support of major branches. As a secondary analysis, we performed a SNP‐based coalescent species tree analysis using SNAPPER (Bryant et al. 2012; Stoltz et al. 2021). For this analysis we filtered the raw GATK output to retain only biallelic SNPs with no missing data, depth ≥ 10, minimum genotype quality of 20 and site quality of 30 and thinned to 1 SNP per 50 kb, resulting in 4085 SNPs. We prepared SNAPPER input XML files using snapp_prep.rb script (https://github.com/mmatschiner/snapp_prep), which employs settings optimized for species divergence time analyses (Stange et al. 2018). We grouped samples to species, subdividing *B. flavifrons* into three geographic groups (eastern fixed red forms from the Southern Rockies, black+red forms from the more centrally located transition region and western Cascade black forms). To estimate divergence times we specified one age constraint based on a prior estimate of divergence time of *B. vagans* from the remaining taxa using a lognormal distribution with an offset of 0, a mean of 5 Ma from Hines (2008) and a standard deviation of the log‐transformed normal distribution of 0.1 in the constraint file to allow for some uncertainty in this time estimate. We ran the snapp_prep.rb script with the ‐a SNAPPER option and randomly retained 2500 SNPs (integer format, homozygotes 0 or 2 at random) with a chain length of 1,000,000 MCMC iterations (store every 250 for 4001 trees) and the XML file was run using BEAST 2.7.5 (Bouckaert et al. 2019). Logs were visualized with TRACER v1.7.2 (Rambaut et al. 2018) to evaluate the MCMC chain stationarity after removing 10% of iterations as burn‐in; all ESS values were > 200 in each run except for the clockRate which had an ESS = 191, which we deemed acceptable as time estimation was a secondary concern. The resulting trees were summarized with TreeAnnotater v2.7.5 and posterior support values and node ages with 95% HPD intervals were visualized with FigTree v1.4.4.

### 4.7 Tests for Introgression

We assessed for potential adaptive introgression by testing for excess allele sharing between *B. flavifrons* populations and outgroups *B. centralis* and *B. vandykei* using the Dsuite program (Malinsky et al. 2021). We provided an initial Newick tree of *(vagans,((flavifronsCascades,flavifronsEast),(centralis,vandykei)))* to automatically guide the set of four possible trios, rooted with *B. vagans* , for analysis. We performed all analyses with the diploid SNP data used for the species tree inference above but only filtered for biallelic sites with a minimum site quality of *Q* ≥ 30 and < 20% missing data, but no genotype quality or depth filters because we employed the genotype‐likelihood approach (–use‐genotype‐probabilities) in Dsuite Dtrios. We used the Dsuite Dtrios program to calculate Patterson's *D* statistic (Durand et al. 2011), with significance estimated using 50 jackknife blocks across the genome. Because true admixture is likely to be reflected in clustered allele sharing (Koppetsch et al. 2024) versus appearing at single sites throughout the genome, we also performed a test for significant clustering of strong admixture informative sites (‐‐ABBAclustering option), using both the sensitive and robust statistical tests (see Koppetsch et al. 2024). We calculated the *f4*‐ratio admixture fraction (Patterson et al. 2012) and, to aid visualization of correlated admixture fractions across the trios in the phylogeny, used the Dsuite Fbranch program to calculate *f*‐branch statistics (Malinsky et al. 2018) using the *f4*‐ratios and the provided rooted tree above using a *p‐*value threshold of 0.01. We used Dinvestigate (20‐SNP windows with 10‐SNP steps) to examine allele sharing statistics in the region of our putative colour locus for the trios.

## Author Contributions

H.M.H., J.D.L. and T.D. designed research. H.M.H. and J.D.L. supervised research. T.D., C.S., H.M.H. and B.M. performed research. J.B.U.K. provided new reagents and analytical tools. T.D., J.D.L. and B.M. analysed data. T.D., H.M.H. and J.D.L. wrote the paper. All authors edited the paper.

## Funding

This project was funded by the National Science Foundation: Division of Environmental Biology, #2126417 to H.M.H. and J.B.U.K. and #2126418 to J.D.L.

## Ethics Statement

The authors have nothing to report.

## Conflicts of Interest

The authors declare no conflicts of interest.

## Supporting information


**Figure S1:** Zoomed in view of k‐mer‐based GWAS. Coverage plot of each sample (top) along with Manhattan plots for the two comparisons (red form [*n* = 41] vs. black form, excluding [*n* = 25] and including Cascade samples [35]). Significant k‐mer positions are indicated in red in GWAS plots and the position of each significant k‐mer for the plot excluding Cascades is indicated with red bars at the top of the coverage plot.
**Figure S2:** Linkage disequilibrium blocks for the variants across the ~20 kb (left) and ~5 kb regions (right) spanning the colour locus. This includes comparisons involving red and black samples excluding (A) and including the Cascade populations (B). Coloured bars indicate ancestral, 7 bp insertion and the repeat regions. Blue dashed lines indicate the peak SNP while the black dashed line parallel to the triangle LD plot indicates this falls in the intron region of the iab8 ncRNA between AbdA and AbdB, with the location of SNPs in the nearest exon indicated (Hines et al. 2025).
**Figure S3:** Manhattan plots from Likelihood ratio test (LRT) genome‐wide association analyses of red and black *Bombus flavifrons* with and without principal component correction and with and without inclusion of the Cascades specimens. The validated tandem duplication on chromosome 18 (CM099917.1) is included in this analysis (as opposed to the main GWAS) and highlighted in blue. The proxy SNP initially detected in our Fisher's exact test is marked as orange. The red dashed line indicates the Bonferroni‐corrected genome‐wide significance threshold and the blue dashed line indicates the suggestive significance threshold (−log_10_(1/*n*)).
**Figure S4:**: SNP variations in the cis‐regulatory region of *Abd‐B* for each of the haplotypes. These are compared to the reference red‐form *B. flavifrons* genome thus tick marks represent variants differing from that sequence. At left are two plots indicating the average colour of all individuals with that haplotype scaled from red to black and the number of individuals of each colour bin (legend of % red at the left). White line/area: deletion; Green line: Insertion; Black and Blue lines: different bases of a SNP from the reference. Eastern, Eastern Oregon (Rockies); Deletion, Black form with deletion haplotypes.
**Figure S5:**: Detailed version of phylogenetic tree analyses of *B. flavifrons* and close relatives using whole genome data. A. A detailed version of the SplitsTree network shown in Figure 4D, with taxon labels (see Table S2), and splits bootstrap support values of major branches of the network. B. Consensus species tree output from SNAPPER (1 million iterations, trees sampled every 250 iterations, removing 10% burn‐in). Branches are labelled with posterior support (all = 1.0), and node bars and text describe the 95% highest posterior density range for estimated divergence times (millions of years before present, mybp).
**Figure S6:**: Test for adaptive introgression between *B. flavifrons* populations and sister species. DSUITE analysis was performed using genotype probabilities using the whole genome samples of *B. flavifrons* (Cascades population and combined eastern population), *B. centralis* and *B. vandykei* , with *B. vagans* as the outgroup, including a graphical summary of the *f‐branch* test, table of statistical results and region‐specific interrogation of the colour‐locus. (A) The provided species tree is displayed along the left and top axes of the matrix. Along the left side, branches (branch *b*; including internal branches displayed as ‘…’) point to a row in the matrix that corresponds to a particular *f‐branch* value (*f*_*b*_) indicated by the colour ramp (grey boxes are not possible comparisons). The *f‐branch* statistics indicate excess allele sharing between branch *b* branches (relative to their sister branch) and the population/species along the top axis. (B) The table shows the four possible triplet comparisons for excess allele sharing for the provided phylogeny (P1, P2 and P3, with the columns containing populations implicated in excess allele sharing indicated in bold). The statistics provided are Patterson's *D* (with *p‐*values indicated using 50 jackknife blocks and for the ‘sensitive’ and ‘robust’ statistical tests for clustering of ABBA‐informative sites (excess allele sharing of P2 and P3) along the genome using the ABBAclustering option), the *f4‐ratio*, and the numbers of sites with the BBAA, ABBA and BABA patterns estimated from genotype likelihoods. (C) Results from Dinterrogate for the admixture statistics *D* and *f*_dM_ using stepping windows (size = 20 SNPs with 10 SNP steps) on scaffold NW_027184763.1 in a 1 Mb region near the putative colour pattern duplication/deletion locus (marked with an arrow), indicating no strong peaks or valleys in the region beyond the variance observed elsewhere along the chromosomes. Note, as an indel polymorphism, the colour locus itself is not included in these analyses.
**Figure S7:** Transcription factor binding sites across different haplotypes of *B. flavifrons* and its sister lineages across the associated genomic region. The Ancestral haplotype row shows the most common ancestral sequence placed into the repeat region to show how TFs vary between the repeat and the ancestral sequence. TF binding sites that are unchanged across samples that have sequence at that location in *B. flavifrons* are labelled in red as ‘Masked’, thus this focused on showing the TF‐binding sites that vary between *B. flavifrons* haplotypes. Vertical dashed lines indicate the SNPs that vary from the *B. flavifrons* genome (red form) for the corresponding haplotypes. Red coloured text indicates a few TF‐binding sites that differ for the California deletion haplotype from the other deletion haplotypes ( *B. vandykei* is identical to the deletion haplotype). The leftmost TFs are added for every haplotype in addition to the sequence alignments that are used to generate this TF mapping to show the binding difference in VG just before the ancestral region begins. CAL, *B. caliginosus*; CENT, *B. centralis* ; DC, Deletion California; VG, *B. vagans* .
**Figure S8:** Enriched functional terms among TF‐binding sites that differ in number with the duplication.
**Table S1:** Specimen information for genomic sequencing for GWAS (A) and for PCR and Sanger sequencing (B). Haplotypes are those used for the haplotype network analysis. AL, Alaska; CA, California; CAN, Canada; CAS, Cascade haplotype; DEL(Gel), samples assessed for indels using gels only (not Sanger sequenced); DEL, Deletion; E[1–12], Eastern haplotypes; ID, Idaho; INS, Insertion; INS_CENT, Centralis insertion; INT, Intermediate; M, male; OR, Oregon; UT, Utah; W, worker (female), WA, Washington; WI, Wisconsin. Coverages represent after alignments. Samples from the first run using 151 bp have been marked with asterisks (*).
**Table S2:** Specimen information for genomic sequencing for samples used for phylogenetic analysis. Read pairs and GB (gigabases) are for post‐trimmed Fastqc samples. The species includes the Eastern vs. Cascades *B. flavifrons* groupings.

## Data Availability

All raw genomic short‐read sequencing data generated for the genome‐wide association study and species tree analysis have been deposited in the NCBI Sequence Read Archive (https://www.ncbi.nlm.nih.gov/sra) under BioProject accession PRJNA1457431 and PRJNA1460806, respectively. The scripts and associated downstream analyses have been deposited in Figshare (https://doi.org/10.6084/m9.figshare.32174169).
