# Supplementary material for: A Cis‐Regulatory Duplication in a Hox Hotspot Implicated in Mimetic Convergence in the Bumble Bee Bombus flavifrons
Source: Mol Ecol. 2026 Sep 7;35(17):e70539. doi: 10.1111/mec.70539 (PMC13547998; doi:10.1111/mec.70539)
Supplement: Supplementary file 1 [file MEC-35-e70539-s001.docx]

**Supplemental Information for:**

**A cis-regulatory duplication in a Hox hotspot implicated in mimetic convergence in the bumble bee *Bombus flavifrons***

Tunç Dabak, Jeffrey D. Lozier, Cecil Smith, Brandon Meadows, Jonathan Berenguer Uhuad Koch, Heather M. Hines

**Table of Contents:**

| **Figure S1** | Page 2 |
| --- | --- |
| **Figure S2** | Page 3 |
| **Figure S3** | Page 4 |
| **Figure S4** | Page 5 |
| **Figure S5** | Page 6 |
| **Figure S6** | Page 7 |
| **Table S1** | Page 8-11 |
| **Table S2** | Page 12-13 |

**Figure S1:** **Zoomed in view of k-mer-based GWAS.** Coverage plot of each sample (top) along with Manhattan plots for the two comparisons [red form (n = 41) vs black form, excluding (n = 25) and including Cascade samples (35)]. Significant k-mer positions are indicated in red in GWAS plots and the position of each significant k-mer for the plot excluding Cascades is indicated with red bars at the top of the coverage plot.

**Figure S2:** **Linkage disequilibrium blocks for the variants across the ~20 kb (left) and ~5 kb regions (right) spanning the color locus.** This includes comparisons involving red and black samples excluding (A) and including the Cascade populations (B). Colored bars indicate ancestral, 7 bp insertion and the repeat regions. Blue dashed lines indicate the peak SNP while the black dashed line parallel to the triangle LD plot indicates this falls in the intron region of the iab8 ncRNA between AbdA and AbdB, with the location of SNPs in the nearest exon indicated (Hines et al. 2025).

**Figure S3: Manhattan plots from Likelihood ratio test (LRT) genome-wide association analyses of red and black *Bombus flavifrons* with and without principal component correction and with and without inclusion of the Cascades specimens.** The validated tandem duplication on chromosome 18 (CM099917.1) is included in this analysis (as opposed to the main GWAS) and highlighted in blue. The proxy SNP initially detected in our Fisher’s exact test is marked as orange. The red dashed line indicates the Bonferroni-corrected genome-wide significance threshold and the blue dashed line indicates the suggestive significance threshold (−log₁₀(1/n)).

**Figure S4**: **SNP variations in the cis-regulatory region of *Abd-B* for each of the haplotypes.** These are compared to the reference red-form *B. flavifrons* genome thus tick marks represent variants differing from that sequence. At left are two plots indicating the average color of all individuals with that haplotype scaled from red to black and the number of individuals of each color bin (legend of % red at the left). White line/area: deletion; Green line: Insertion; Black and Blue lines: different bases of a SNP from the reference. Eastern = Eastern Oregon (Rockies), Deletion = Black form with deletion haplotypes.

**Figure S5:** **Detailed version of phylogenetic tree analyses of *B. flavifrons* and close relatives using whole genome data.** A. A detailed version of the SplitsTree network shown in Fig. 4D, with taxon labels (see Supplementary Table 2), and splits bootstrap support values of major branches of the network. B. Consensus species tree output from SNAPPER (1 million iterations, trees sampled every 250 iterations, removing 10% burn-in). Branches are labeled with posterior support (all = 1.0), and node bars and text describe the 95% highest posterior density range for estimated divergence times (millions of years before present, mybp).

**Figure S6**: **Test for adaptive introgression between *B. flavifrons* populations and sister species.** DSUITE analysis was performed using genotype probabilities using the whole genome samples of *B. flavifrons* (Cascades population and combined eastern population), *B. centralis,* and *B. vandykei*, with *B. vagans* as the outgroup, including a graphical summary of the *f-branch* test, table of statistical results, and region-specific interrogation of the color-locus. A) The provided species tree is displayed along the left and top axes of the matrix. Along the left side, branches (branch *b*; including internal branches displayed as “…”) point to a row in the matrix that corresponds to a particular *f-branch* value (f_b_) indicated by the color ramp (gray boxes are not possible comparisons). The *f-branch* statistics indicate excess allele sharing between branch *b* branches (relative to their sister branch) and the population/species along the top axis. B) The table shows the four possible triplet comparisons for excess allele sharing for the provided phylogeny (P1, P2, and P3, with the columns containing populations implicated in excess allele sharing indicated in bold). The statistics provided are Patterson’s *D* (with *p-*values indicated using 50 jackknife blocks and for the “sensitive” and “robust” statistical tests for clustering of ABBA-informative sites (excess allele sharing of P2 and P3) along the genome using the ABBAclustering option), the *f4-ratio*, and the numbers of sites with the BBAA, ABBA, and BABA patterns estimated from genotype likelihoods. C) Results from Dinterrogate for the admixture statistics *D* and *f*_dM_ using stepping windows (size = 20 SNPs with 10 SNP steps) on scaffold NW_027184763.1 in a 1 Mb region near the putative color pattern duplication/deletion locus (marked with an arrow), indicating no strong peaks or valleys in the region beyond the variance observed elsewhere along the chromosomes. Note, as an indel polymorphism, the color locus itself is not included in these analyses.

**Figure S7: Transcription factor binding sites across different haplotypes of *B. flavifrons* and its sister lineages** **across the associated genomic region.** The Ancestral haplotype row shows the most common ancestral sequence placed into the repeat region to show how TFs vary between the repeat and the ancestral sequence. TF binding sites that are unchanged across samples that have sequence at that location in *B. flavifrons* are labeled in red as “Masked”, thus this focused on showing the TF-binding sites that vary between *B. flavifrons* haplotypes. Vertical dashed lines indicate the SNPs that vary from the *B. flavifrons* genome (red form) for the corresponding haplotypes. Red colored text indicates a few TF-binding sites that differ for the California deletion haplotype from the other deletion haplotypes (*B. vandykei* is identical to the deletion haplotype). The leftmost TFs are added for every haplotype in addition to the sequence alignments that are used to generate this TF mapping to show the binding difference in VG just before the ancestral region begins. CAL = *B.caliginosus*, VG = *B. vagans*, DC = Deletion California, CENT = *B. centralis*.

**Figure S8: Enriched functional terms among TF-binding sites that differ in number with the duplication.**

**Table S1.** **Specimen information for genomic sequencing for GWAS (A) and for PCR and Sanger sequencing (B).** Haplotypes are those used for the haplotype network analysis. INT = Intermediate, OR = Oregon, WA = Washington, CA = California, UT = Utah, WI = Wisconsin, ID = Idaho, CAN = Canada, AL = Alaska, INS = Insertion, DEL= Deletion, M = Male, W = Worker (female), CAS = Cascade Haplotype, INS_CENT = Centralis Insertion, E[1-12] = Eastern Haplotypes, DEL(Gel) = samples assessed for indels using gels only (not Sanger sequenced). Coverages represent after alignments. Samples from the first run using 151bp have been marked with asterisks (*).

A. **GWAS Samples**

| **Sample** | **Latitude** | | **Longitude** | **%Red** | **Color** | **Sex** | **Species** | **State** | **Haplotype** | **Read Pairs** | | **Coverage** |
| --- | --- | --- | --- | --- | --- | --- | --- | --- | --- | --- | --- | --- |
| R23 | | 45.07601 | -117.507 | 100 | R | M | *centralis* | OR | INS_CENT | 18446100 | | 14.22X |
| B3 | | 46.20972 | -116.842 | 0 | B | M | *flavifrons* | OR | DEL(Gel) | 18269488 | | 14.09X |
| B4 | | 45.69448 | -117.284 | 0 | B | M | *flavifrons* | OR | DEL | 30726478 | | 23.70X |
| B5 | | 45.69448 | -117.284 | 0 | B | M | *flavifrons* | OR | E_1 | 21638106 | | 16.69X |
| B10 | | 45.27116 | -117.122 | 0 | B | M | *flavifrons* | OR | DEL(Gel) | 26517658 | | 20.45X |
| B12 | | 45.31245 | -117.306 | 0 | B | M | *flavifrons* | OR | DEL(Gel) | 21200073 | | 16.35X |
| B13 | | 45.31245 | -117.306 | 0 | B | M | *flavifrons* | OR | DEL(Gel) | 18325727 | | 14.13X |
| B14 | | 45.42162 | -117.815 | 0 | B | M | *flavifrons* | OR | DEL(Gel) | 18393595 | | 14.18X |
| B15 | | 45.40414 | -117.707 | 0 | B | M | *flavifrons* | OR | DEL | 20861593 | | 16.09X |
| B17 | | 45.27188 | -117.387 | 0 | B | M | *flavifrons* | OR | E_9 | 19528123 | | 15.06X |
| B18 | | 45.16945 | -117.078 | 0 | B | M | *flavifrons* | OR | DEL | 19229634 | | 14.83X |
| B19 | | 45.16945 | -117.078 | 0 | B | M | *flavifrons* | OR | DEL | 19277507 | | 14.87X |
| B20 | | 44.99237 | -117.156 | 0 | B | M | *flavifrons* | OR | DEL | 18228865 | | 14.06X |
| B21 | | 44.99237 | -117.156 | 0 | B | M | *flavifrons* | OR | E_8 | 19459710 | | 15.01X |
| B22 | | 45.07601 | -117.507 | 0 | B | M | *flavifrons* | OR | E_7 | 18349157 | | 14.15X |
| B23 | | 45.07601 | -117.507 | 0 | B | M | *flavifrons* | OR | Alaska_E_11 | 20978264 | | 16.18X |
| B24 | | 45.49099 | -118.152 | 0 | B | M | *flavifrons* | OR | DEL | 23650149 | | 18.24X |
| JK37* | | 44.68815 | -118.024 | 0 | B | M | *flavifrons* | OR | DEL | 12631574 | | 9.74X |
| JKAL36* | | 44.68815 | -118.024 | 0 | B | M | *flavifrons* | OR | DEL | 16209867 | | 12.50X |
| TDB001* | | 43.29669 | -122.909 | 0 | B | M | *flavifrons* | OR | CAS_0 | 13687061 | | 10.55X |
| TDB002* | | 44.79286 | -121.838 | 0 | B | M | *flavifrons* | OR | CAS_0 | 13871398 | | 10.70X |
| TDB003* | | 45.50375 | -122.684 | 0 | B | M | *flavifrons* | OR | CAS_1 | 16628247 | | 12.82X |
| TDB004* | | 47.26352 | -120.698 | 0 | B | M | *flavifrons* | WA | CAS_0 | 26892970 | | 20.74X |
| TDB009* | | 45.64764 | -118.128 | 0 | B | M | *flavifrons* | OR | E_5 | 13787878 | | 10.63X |
| TDB010* | | 45.7019 | -118.035 | 0 | B | M | *flavifrons* | OR | E_1 | 14131273 | | 10.90X |
| TDB011* | | 45.64764 | -118.128 | 0 | B | M | *flavifrons* | OR | DEL | 13753557 | | 10.61X |
| TDB012* | | 45.7019 | -118.035 | 0 | B | M | *flavifrons* | OR | DEL | 13447601 | | 10.37X |
| TDB013* | | 45.27709 | -117.136 | 0 | B | M | *flavifrons* | OR | DEL | 26517891 | | 20.45X |
| TDB016* | | 46.14787 | -116.787 | 0 | B | M | *flavifrons* | OR | DEL | 11865922 | | 9.15X |
| TDB017* | | 45.30289 | -121.794 | 0 | B | M | *flavifrons* | OR | CAS_0 | 14273322 | | 11.01X |
| TDB018* | | 43.90778 | -122.197 | 0 | B | M | *flavifrons* | OR | CAS_0 | 30855962 | | 23.79X |
| TDB022* | | 43.29669 | -122.909 | 0 | B | M | *flavifrons* | OR | CAS_0 | 12272288 | | 9.46X |
| TDB023* | | 45.73093 | -117.277 | 0 | B | M | *flavifrons* | OR | DEL | 14447148 | | 11.14X |
| TDB019* | | 45.50375 | -122.684 | 0 | B | M | *flavifrons* | OR | CAS_0 | 16864563 | | 13.01X |
| TDB020* | | 43.90778 | -122.197 | 0 | B | M | *flavifrons* | OR | INS(Gel) | 15011402 | | 11.58X |
| TDB021* | | 45.30289 | -121.794 | 0 | B | M | *flavifrons* | OR | CAS_0 | 15788343 | | 12.18X |
| R1 | | 46.16734 | -116.735 | 90 | R | M | *flavifrons* | OR | E_1 | 19879979 | | 15.33X |
| R2 | | 46.14798 | -116.787 | 90 | R | M | *flavifrons* | OR | E_1 | 22072996 | | 17.02X |
| R3 | | 46.19323 | -116.807 | 90 | R | M | *flavifrons* | OR | E_4 | 17807401 | | 13.73X |
| R4 | | 46.19323 | -116.807 | 90 | R | M | *flavifrons* | OR | E_1 | 23374861 | | 18.03X |
| R6 | | 45.28285 | -117.208 | 90 | R | M | *flavifrons* | OR | E_1 | 17065478 | | 13.16X |
| R7 | | 45.28285 | -117.208 | 90 | R | M | *flavifrons* | OR | E_6 | 20713127 | | 15.97X |
| R8 | | 45.27526 | -117.138 | 90 | R | M | *flavifrons* | OR | E_1 | 16522608 | | 12.74X |
| R10 | | 45.27116 | -117.122 | 90 | R | M | *flavifrons* | OR | INS(Gel) | 20081172 | | 15.49X |
| R11 | | 45.27116 | -117.122 | 90 | R | M | *flavifrons* | OR | INS(Gel) | 21502126 | | 16.58X |
| R12 | | 45.31245 | -117.306 | 90 | R | M | *flavifrons* | OR | E_1 | 18529829 | | 14.29X |
| R13 | | 45.31245 | -117.306 | 90 | R | M | *flavifrons* | OR | INS(Gel) | 17489337 | | 13.49X |
| R14 | | 45.42162 | -117.815 | 90 | R | M | *flavifrons* | OR | INS(Gel) | 17289082 | | 13.33X |
| R15 | | 45.47171 | -117.559 | 90 | R | M | *flavifrons* | OR | INS(Gel) | 17823402 | | 13.74X |
| R16 | | 45.31674 | -117.401 | 90 | R | M | *flavifrons* | OR | INS(Gel) | 18356499 | | 14.16X |
| R17 | | 45.27188 | -117.387 | 90 | R | M | *flavifrons* | OR | E_1 | 22447929 | | 17.31X |
| R18 | | 45.16945 | -117.078 | 90 | R | M | *flavifrons* | OR | E_6 | 21146782 | | 16.31X |
| R19 | | 45.16945 | -117.078 | 90 | R | M | *flavifrons* | OR | E_1 | 17644161 | | 13.61X |
| R20 | | 44.99237 | -117.156 | 90 | R | M | *flavifrons* | OR | E_1 | 21712386 | | 16.74X |
| R21 | | 44.99237 | -117.156 | 90 | R | M | *flavifrons* | OR | E_1 | 17260562 | | 13.31X |
| R22 | | 45.07601 | -117.507 | 90 | R | M | *flavifrons* | OR | E_3 | 19293815 | | 14.88X |
| R24 | | 45.49099 | -118.152 | 90 | R | M | *flavifrons* | OR | E_1 | 18994748 | | 14.65X |
| R28 | | 45.27188 | -117.387 | 90 | R | M | *flavifrons* | OR | E_5 | 14855134 | | 11.46X |
| R31 | | 46.20972 | -116.842 | 90 | R | M | *flavifrons* | OR | E_1 | 21285300 | | 16.41X |
| JKMIR022* | | 40.602 | -110.968 | 90 | R | M | *flavifrons* | UT | INS(Gel) | 13869348 | | 10.70X |
| MIR001A* | | 40.4205 | -110.531 | 90 | R | M | *flavifrons* | UT | INS(Gel) | 25396582 | | 19.58X |
| TDR002* | | 48.68074 | -118.905 | 90 | R | M | *flavifrons* | WA | E_1 | 15149548 | | 11.68X |
| TDR003* | | 48.68074 | -118.905 | 90 | R | M | *flavifrons* | WA | E_1 | 12838528 | | 9.90X |
| TDR005* | | 46.63481 | -114.43 | 90 | R | M | *flavifrons* | ID | E_1 | 11755636 | | 9.07X |
| TDR006* | | 45.04024 | -116.332 | 90 | R | M | *flavifrons* | ID | E_1 | 19715076 | | 15.20X |
| TDR007* | | 45.04024 | -116.332 | 90 | R | M | *flavifrons* | ID | E_4 | 13308024 | | 10.26X |
| TDR008* | | 45.00777 | -117.192 | 90 | R | M | *flavifrons* | OR | E_1 | 14254067 | | 10.99X |
| TDR009* | | 45.27709 | -117.136 | 90 | R | M | *flavifrons* | OR | INS(Gel) | 16026086 | | 12.36X |
| TDR010* | | 45.73093 | -117.277 | 90 | R | M | *flavifrons* | OR | INS(Gel) | 19424401 | | 14.98X |
| TDR011* | | 45.64764 | -118.128 | 90 | R | M | *flavifrons* | OR | INS(Gel) | 15758525 | | 12.15X |
| TDR012* | | 45.64764 | -118.128 | 90 | R | M | *flavifrons* | OR | INS(Gel) | | 13026442 | 10.05X |
| TDR016* | | 46.63481 | -114.43 | 90 | R | M | *flavifrons* | ID | INS(Gel) | | 12382685 | 9.55X |
| TDR017* | | 46.23098 | -115.416 | 90 | R | M | *flavifrons* | ID | INS(Gel) | | 16298501 | 12.57X |
| TDR018* | | 48.59607 | -118.644 | 90 | R | M | *flavifrons* | WA | INS(Gel) | | 14509439 | 11.19X |
| TDR019* | | 46.23098 | -115.416 | 90 | R | M | *flavifrons* | ID | INS | | 13955840 | 10.76X |
| TDR021* | | 45.27709 | -117.136 | 90 | R | M | *flavifrons* | OR | INS(Gel) | | 15563627 | 12.00X |
| TDR025* | | 45.64764 | -118.128 | 90 | R | M | *flavifrons* | OR | INS(Gel) | | 13481895 | 10.40X |

B. **PCR and Sanger Samples**

| **Sample** | **Latitude** | **Longitude** | **%Red** | **Color** | **Sex** | **Species** | **State** | **Haplotype** |
| --- | --- | --- | --- | --- | --- | --- | --- | --- |
| Flav_M_Int1 | 45.73093 | -117.27666 | 35 | INT | M | *flavifrons* | OR | E_7 |
| Flav_M_Int2 | 45.73093 | -117.27666 | 35 | INT | M | *flavifrons* | OR | E_1 |
| Flav_M_Int3 | 45.7019 | -118.03515 | 25 | INT | M | *flavifrons* | OR | E_7 |
| Flav_M_Int4 | 45.73093 | -117.27666 | 35 | INT | M | *flavifrons* | OR | E_7 |
| Flav_M_Int5 | 45.27709 | -117.13616 | 37 | INT | M | *flavifrons* | OR | E_1 |
| Flav_M_Int6 | 48.68074 | -118.90534 | 45 | INT | M | *flavifrons* | WA | E_1 |
| Flav_M_Int7 | 45.73093 | -117.27666 | 40 | INT | M | *flavifrons* | OR | E_1 |
| Flav_M_Int8 | 45.29559 | -117.39569 | 50 | INT | M | *flavifrons* | OR | E_1 |
| Flav_M_Int9 | 44.31334 | -118.67955 | 50 | INT | M | *flavifrons* | OR | E_5 |
| Flav_M_Int10 | 45.73093 | -117.27666 | 50 | INT | M | *flavifrons* | OR | E_1 |
| Flav_M_Int11 | 45.73093 | -117.27666 | 50 | INT | M | *flavifrons* | OR | E_1 |
| Flav_M_Int12 | 45.40414 | -117.706573 | 40 | INT | M | *flavifrons* | OR | E_1 |
| Flav_M_Int13 | 46.16734 | -116.73507 | 60 | INT | M | *flavifrons* | OR | E_6 |
| Flav_M_Int14 | 45.27526 | -117.13765 | 42 | INT | M | *flavifrons* | OR | E_1 |
| Flav_M_Int15 | 45.31674 | -117.400834 | 60 | INT | M | *flavifrons* | OR | E_1 |
| Flav_M_Int16 | 45.69448 | -117.283701 | 50 | INT | M | *flavifrons* | OR | E_4 |
| Flav_M_Int17 | 45.28285 | -117.208383 | 70 | INT | M | *flavifrons* | OR | E_1 |
| Flav_M_Int18 | 46.19323 | -116.80677 | 70 | INT | M | *flavifrons* | OR | E_2 |
| Flav_M_Int19 | 45.69996 | -117.297803 | 40 | INT | M | *flavifrons* | OR | INS(Gel) |
| Flav_M_Int20 | 45.27116 | -117.122435 | 40 | INT | M | *flavifrons* | OR | E_6 |
| Flav_M_Black1 | 46.16734 | -116.73507 | 0 | B | M | *flavifrons* | OR | DEL(Gel) |
| Flav_M_Black3 | 46.14798 | -116.78676 | 0 | B | M | *flavifrons* | OR | DEL(Gel) |
| Flav_M_Black4 | 46.19323 | -116.80677 | 0 | B | M | *flavifrons* | OR | DEL(Gel) |
| Flav_M_Black5 | 46.19323 | -116.80677 | 0 | B | M | *flavifrons* | OR | DEL(Gel) |
| Flav_M_Black6 | 46.19323 | -116.80677 | 0 | B | M | *flavifrons* | OR | DEL(Gel) |
| Flav_M_Black8 | 45.69448 | -117.283701 | 0 | B | M | *flavifrons* | OR | DEL(Gel) |
| Flav_M_Black9 | 45.69996 | -117.297803 | 0 | B | M | *flavifrons* | OR | DEL(Gel) |
| Flav_M_Black10 | 45.68687 | -117.35989 | 0 | B | M | *flavifrons* | OR | DEL(Gel) |
| Flav_M_Black11 | 45.68687 | -117.35989 | 0 | B | M | *flavifrons* | OR | DEL(Gel) |
| Flav_M_Black13 | 45.27526 | -117.13765 | 0 | B | M | *flavifrons* | OR | DEL(Gel) |
| Flav_M_Black14 | 45.27526 | -117.13765 | 0 | B | M | *flavifrons* | OR | DEL(Gel) |
| Flav_M_Black15 | 45.69996 | -117.297803 | 0 | B | M | *flavifrons* | OR | DEL(Gel) |
| Flav_M_Black16 | 45.27116 | -117.122435 | 0 | B | M | *flavifrons* | OR | DEL(Gel) |
| Flav_M_Black17 | 45.31245 | -117.306367 | 0 | B | M | *flavifrons* | OR | DEL(Gel) |
| Flav_M_Black18 | 45.31245 | -117.306367 | 0 | B | M | *flavifrons* | OR | DEL(Gel) |
| Flav_M_Black19 | 45.40414 | -117.706573 | 0 | B | M | *flavifrons* | OR | DEL(Gel) |
| Flav_M_Black21 | 45.31674 | -117.400834 | 0 | B | M | *flavifrons* | OR | DEL(Gel) |
| Flav_M_Black22 | 45.31674 | -117.400834 | 0 | B | M | *flavifrons* | OR | DEL(Gel) |
| Flav_M_Black23 | 45.31674 | -117.400834 | 0 | B | M | *flavifrons* | OR | DEL(Gel) |
| Flav_M_Black24 | 45.31674 | -117.400834 | 0 | B | M | *flavifrons* | OR | DEL(Gel) |
| Flav_M_Black25 | 45.27188 | -117.386967 | 0 | B | M | *flavifrons* | OR | DEL(Gel) |
| Flav_M_Black26 | 45.16945 | -117.077889 | 0 | B | M | *flavifrons* | OR | DEL(Gel) |
| Flav_M_Black27 | 45.16945 | -117.077889 | 0 | B | M | *flavifrons* | OR | DEL(Gel) |
| Flav_M_Black28 | 44.99237 | -117.156253 | 0 | B | M | *flavifrons* | OR | DEL(Gel) |
| Flav_M_Black29 | 44.99237 | -117.156253 | 0 | B | M | *flavifrons* | OR | E_1 |
| fW_SC095 | 38.69325 | -119.990778 | 0 | B | M | *flavifrons* | CA | DEL_CAL |
| FB04 | 44.99237 | -117.156253 | 0 | B | M | *flavifrons* | OR | E_11 |
| FB05 | 45.07601 | -117.507028 | 0 | B | M | *flavifrons* | OR | E_11 |
| FB06 | 45.07601 | -117.507028 | 0 | B | M | *flavifrons* | OR | E_7 |
| FB07 | 45.07601 | -117.507028 | 0 | B | M | *flavifrons* | OR | E_6 |
| FB10 | 45.27526 | -117.13765 | 0 | B | M | *flavifrons* | OR | E_8 |
| FB15 | 45.49099 | -118.151512 | 0 | B | M | *flavifrons* | OR | E_10 |
| FB01 | 45.31674 | -117.400834 | 0 | B | M | *flavifrons* | OR | DEL(Gel) |
| FB02 | 45.31245 | -117.306367 | 0 | B | M | *flavifrons* | OR | DEL(Gel) |
| FB03 | 44.99237 | -117.156253 | 0 | B | M | *flavifrons* | OR | DEL(Gel) |
| FB08 | 45.27526 | -117.13765 | 0 | B | M | *flavifrons* | OR | DEL(Gel) |
| FB09 | 45.27526 | -117.13765 | 0 | B | M | *flavifrons* | OR | DEL(Gel) |
| FB11 | 45.49099 | -118.151512 | 0 | B | M | *flavifrons* | OR | DEL(Gel) |
| FB12 | 45.49099 | -118.151512 | 0 | B | M | *flavifrons* | OR | DEL(Gel) |
| FB13 | 45.49099 | -118.151512 | 0 | B | M | *flavifrons* | OR | DEL(Gel) |
| FB14 | 45.49099 | -118.151512 | 0 | B | M | *flavifrons* | OR | DEL(Gel) |
| Flav_W_Red1 | 46.16734 | -116.73507 | 95 | R | W | *flavifrons* | OR | E_1 |
| Flav_W_Red2 | 46.16734 | -116.73507 | 90 | R | W | *flavifrons* | OR | Het_E1_6 |
| Flav_W_Red3 | 46.16734 | -116.73507 | 98 | R | W | *flavifrons* | OR | E_6 |
| Flav_W_Red4 | 45.27116 | -117.122435 | 98 | R | W | *flavifrons* | OR | E_1 |
| Flav_W_Red5 | 45.31245 | -117.306367 | 98 | R | W | *flavifrons* | OR | Het_E1_6 |
| Flav_W_Red6 | 40.48565 | -111.63842 | 100 | R | W | *flavifrons* | UT | E_1 |
| fF_Alb | 51.09539 | -115.059694 | 99 | R | W | *flavifrons* | CAN | E_12 |
| fF_2005_02 | 64.86669 | -147.664861 | 100 | R | W | *flavifrons* | AL | E_11 |
| Flav_W_Int1 | 45.69996 | -117.297803 | 50 | INT | W | *flavifrons* | OR | E_1 |
| Flav_W_Int2 | 45.27526 | -117.13765 | 60 | INT | W | *flavifrons* | OR | Het_E6_Del |
| Flav_W_Int3 | 45.27526 | -117.13765 | 50 | INT | W | *flavifrons* | OR | Het_E1_Del |
| Flav_W_Int4 | 45.45549 | -117.733227 | 65 | INT | W | *flavifrons* | OR | Het_E1_7 |
| Flav_W_Int5 | 40.51667 | -121.49402 | 40 | INT | W | *flavifrons* | CA | DEL |
| Flav_W_Int6 | 45.16945 | -117.077889 | 50 | INT | W | *flavifrons* | OR | Het_E6_Del |
| Flav_W_Black1 | 45.68687 | -117.35989 | 0 | B | W | *flavifrons* | OR | Het_E7_Del |
| Flav_W_Black2 | 45.47171 | -117.55863 | 0 | B | W | *flavifrons* | OR | Het_E7_Del |
| Flav_W_Black3 | 45.49099 | -118.151512 | 0 | B | W | *flavifrons* | OR | DEL |
| Flav_W_Black4 | 45.07601 | -117.507028 | 0 | B | W | *flavifrons* | OR | DEL |
| Flav_W_Black5 | 40.51667 | -121.49402 | 0 | B | W | *flavifrons* | CA | DEL |
| Flav_W_Black6 | 44.79286 | -121.83786 | 0 | B | W | *flavifrons* | OR | CAS_0 |
| fW_SC017 | 38.69325 | -119.990778 | 0 | B | W | *flavifrons* | CA | DEL_CAL |
| vagans | 43.08848 | -89.41707 | 0 | B | M | *vagans* | WI | Del_Vagans |
| caliginosus | 39.62908 | -123.465389 | 0 | B | M | *caliginosus* | CA | Del_CALIG |
| vandykei | Unknown | Unknown | 0 | B | M | *vandykei* | CA | Del_VANDY |
| cenw_2003_04 | 46.85517 | -120.482506 | 100 | R | W | *centralis* | WA | INS_CENT |
| HH15_3903 | 45.64764 | -118.12817 | 100 | R | M | *centralis* | ID | INS_CENT2 |

**Table S2.** **Specimen information for genomic sequencing for samples used for phylogenetic analysis.** Read pairs and GB (gigabases) are for post-trimmed Fastqc samples. The species includes the Eastern vs. Cascades *B. flavifrons* groupings.

| **Sample ID** | **Species** | **Color** | **State** | **Latitude** | **Longitude** | **Read Pairs (x10^6^)** | **GB** | **Mean VCF Depth** |
| --- | --- | --- | --- | --- | --- | --- | --- | --- |
| BRL1209calig | *caliginosus* | Black | Oregon | 42.827 | -124.525 | 17.029 | 4.856 | 14.8183 |
| BRL1204_caligin | *caliginosus* | Black | Oregon | 42.269 | -124.400 | 20.518 | 6.010 | 16.4842 |
| BRL1201_caligin | *caliginosus* | Black | Oregon | 42.141 | -124.353 | 30.976 | 8.937 | 23.2393 |
| HH153602_1_caliginosus | *caliginosus* | Black | Oregon | 45.770 | -123.948 | 18.635 | 5.553 | 13.1789 |
| BRL1216_central | *centralis* | Red | Oregon | 45.009 | -119.690 | 23.366 | 6.367 | 17.7711 |
| HH150303_1_centralis | *centralis* | Red | Oregon | 42.750 | -118.677 | 17.523 | 5.217 | 14.3702 |
| BRL1279_central | *centralis* | Red | Idaho | 45.856 | -116.081 | 28.892 | 8.196 | 21.7407 |
| CO08x0040cen | *centralis* | Red | Colorado | 39.156 | -105.156 | 20.472 | 5.822 | 13.0801 |
| SD08x0569cen | *centralis* | Red | South Dakota | 44.389 | -103.622 | 14.088 | 4.069 | 11.3015 |
| HHflav1_flavifrons | *flavifrons (Cascades)* | Black | Oregon | 44.504 | -123.551 | 20.301 | 6.046 | 14.8658 |
| BRL1225_flavifr | *flavifrons (Cascades* | Black | Washington | 48.103 | -123.416 | 18.230 | 5.295 | 13.887 |
| HH151803_1_flavifrons | *flavifrons (Cascades* | Black | California | 42.000 | -122.770 | 15.472 | 4.616 | 13.1018 |
| HH152605_1_flavifrons | *flavifrons (Cascades* | Black | Oregon | 43.908 | -122.197 | 17.133 | 5.111 | 13.6424 |
| BRL0268_flavifr | *flavifrons (Cascades* | Black | Oregon | 44.224 | -121.872 | 16.481 | 4.794 | 12.9631 |
| BRL0216_flavifr | *flavifrons (Cascades* | Black | Oregon | 45.320 | -121.622 | 17.192 | 4.962 | 13.9775 |
| BRL1219B_flav | *flavifrons (Eastern)* | Black | Oregon | 44.952 | -119.708 | 25.899 | 7.536 | 21.135 |
| BRL1259_flav_re | *flavifrons (Eastern)* | Red | Washington | 48.608 | -118.478 | 16.853 | 4.903 | 13.0256 |
| JBUK782_flav_re | *flavifrons (Eastern)* | Black | Oregon | 44.688 | -118.024 | 16.617 | 4.858 | 13.7659 |
| BRL1284_flavifr | *flavifrons (Eastern)* | Red | Washington | 48.654 | -117.485 | 16.663 | 4.759 | 12.758 |
| BRL1263_flavifr | *flavifrons (Eastern)* | Black | Oregon | 45.277 | -117.136 | 20.533 | 5.989 | 16.2565 |
| HH15_0203_1_flavifrons | *flavifrons (Eastern)* | Red | Idaho | 43.783 | -116.086 | 14.695 | 4.371 | 12.4631 |
| BRL1275flavi | *flavifrons (Eastern)* | Red | Montana | 48.454 | -115.773 | 19.857 | 5.605 | 17.4707 |
| JBUK436Bflav | *flavifrons (Eastern)* | Red | Utah | 40.605 | -111.518 | 17.785 | 5.214 | 17.2246 |
| CO08x0126_flavi | *flavifrons (Eastern)* | Red | Colorado | 40.023 | -105.514 | 26.467 | 7.538 | 19.3909 |
| BRL1260_vagans | *vagans* | Black | Washington | 48.062 | -120.026 | 17.920 | 5.145 | 14.3568 |
| BRL1286_vagans | *vagans* | Black | Washington | 48.654 | -117.485 | 41.823 | 11.909 | 35.9747 |
| HH151805_1_vandykei | *vandykei* | Black | California | 42.000 | -122.770 | 15.422 | 4.590 | 12.4516 |
| BRL0214_vandyke | *vandykei* | Black | Oregon | 45.335 | -121.663 | 22.246 | 6.582 | 17.9269 |
| BRL1329_vandyke | *vandykei* | Black | Washington | 47.264 | -120.698 | 28.264 | 7.946 | 21.7236 |
| BRL1331_vandyke | *vandykei* | Black | Washington | 48.580 | -120.479 | 31.358 | 8.734 | 23.6023 |
| HH150602_1_vandykei | *vandykei* | Black | California | 41.546 | -120.247 | 22.993 | 6.852 | 18.7514 |
